# Invariância da *Escala Brasileira de Insegurança Alimentar*: análise de grupos demográficos a partir da *Pesquisa Nacional por Amostra de Domicílios*

**DOI:** 10.1590/0102-311XPT115125

**Published:** 2026-07-24

**Authors:** Vanessa Guimarães Cezimbra, João Luiz Bastos, Michael Eduardo Reichenheim

**Affiliations:** 1 Universidade Federal de Santa Catarina, Florianópolis, Brasil.; 2 Universidade do Estado do Rio de Janeiro, Rio de Janeiro, Brasil.

**Keywords:** Insegurança Alimentar, Iniquidade em Saúde, População Rural, População Urbana, Food Security, Health Inequities, Rural Population, Urban Population, Inseguridad Alimentaria, Iniquidad en Salud, Población Rural, Población Urbana

## Abstract

A *Escala Brasileira de Insegurança Alimentar* (EBIA) tem sido amplamente empregada em inquéritos populacionais para mensurar a insegurança alimentar no Brasil. Embora suas propriedades psicométricas estejam bem estabelecidas, poucos estudos avaliaram sua invariância de mensuração entre diferentes contextos territoriais, o que é essencial para garantir a validade de comparações regionais. Este estudo investigou a invariância da EBIA em relação à situação urbana/rural, macrorregiões e estados do Brasil, com base nos dados da *Pesquisa Nacional por Amostra de Domicílios* (PNAD) de 2013. Foram analisados os oito itens gerais/de adulto da EBIA, por meio do método de alinhamento, em três partições independentes da amostra, totalizando 116.543 domicílios. Os resultados indicaram que a EBIA apresentou invariância entre áreas urbanas e rurais e invariância parcial entre as regiões e entre estados. Esses achados reforçam a validade do uso da escala para monitorar e comparar a insegurança alimentar em diferentes regiões do país.

## Abstract (en)

Population surveys have widely used the *Brazilian Food Insecurity Scale* (EBIA, acronym in Portuguese) to measure food insecurity in the country. Despite its well-established psychometric properties, few studies have evaluated its measurement invariance between territories, which is essential to ensure the validity of regional comparisons. This study investigated EBIA invariance regarding urban/rural areas, macro-regions, and Brazilian states based on data from the 2013 *Brazilian National Household Sample Survey* (PNAD, acronym in Portuguese). It analyzed the eight general/adult EBIA items by alignment in three independent partitions of the sample (116,543 households). The results indicated that the EBIA showed invariance between urban and rural areas and partial invariance between regions and between states. These findings reinforce the validity of the EBIA to monitor and compare food insecurity across Brazil.

## Resumen

La *Escala Brasileña de Inseguridad Alimentaria* (EBIA) se ha utilizado ampliamente en encuestas poblacionales para medir la inseguridad alimentaria en Brasil. Aunque sus propiedades psicométricas están bien establecidas, pocos estudios han evaluado la invariancia de su medición en diferentes contextos territoriales, lo cual es esencial para asegurar la validez de las comparaciones regionales. Este estudio investigó la invariancia del EBIA en relación con la ubicación urbana/rural, las macrorregiones y los estados de Brasil, con base en datos de la *Encuesta Nacional por Muestra de Domicilios* (PNAD, por sus siglas en portugués) del 2013. Los ocho ítems generales/para adultos de la EBIA se analizaron utilizando el método de alineación en tres particiones independientes de la muestra, que totalizaron 116.543 hogares. Los resultados indicaron que el EBIA mostró invariancia entre áreas urbanas y rurales, e invariancia parcial entre las regiones y entre estados. Estos hallazgos refuerzan la validez del uso de la escala para monitorear y comparar la inseguridad alimentaria en diferentes regiones del país.

## Introdução

A violação do direito humano à alimentação adequada, entendida como a falta de acesso a alimentos de qualidade e em quantidades satisfatórias, pode levar indivíduos e famílias à situação de insegurança alimentar ^1^ ^,^ ^2^. No Brasil, houve uma redução do número de domicílios em situação de insegurança alimentar ^3^. Esse progresso foi reconhecido pela Organização das Nações Unidas para Alimentação e Agricultura (FAO), resultando na saída do país do Mapa da Fome em 2014. No entanto, a partir de 2015, essa tendência inverteu e se intensificou durante a pandemia de COVID-19. No final de 2021 e início de 2022, quase dois terços (58,7%) dos domicílios brasileiros se encontravam em algum grau de insegurança alimentar ^4^. Segundo projeções baseando-se na *Pesquisa Nacional por Amostra de Domicílios* (PNAD) *Contínua*, o país tinha 27,6% dos seus lares em insegurança alimentar em 2023 ^5^. Os dados desse inquérito sugerem que as desigualdades regionais no acesso à alimentação adequada permanecem significativas. As regiões Norte (7,7%) e Nordeste (6,2%) continuam apresentando as maiores prevalências de insegurança alimentar grave, já as regiões Sudeste (2,9%) e Sul (2%) registram os menores percentuais. Além disso, a proporção de domicílios em insegurança alimentar grave era maior nas áreas rurais (5,5%) do que nas áreas urbanas (3,9%) ^5^.

É fundamental considerar que esse cenário depende de uma mensuração concreta da prevalência de insegurança alimentar, permitindo uma compreensão mais aprofundada da fome, das suas iniquidades e de seus determinantes. Informações válidas e confiáveis acerca desse problema são essenciais para formular e avaliar políticas que garantam o direito à alimentação adequada ^6^ ^,^ ^7^. A insegurança alimentar tem sido mensurada, de forma satisfatória, há décadas por instrumentos como o Módulo da Pesquisa sobre Segurança Alimentar dos Agregados Familiares dos Estados Unidos (*US Household Food Security Survey Module*; HFSSM, acrônimo em inglês), o que permite que os dados reflitam com validade e precisão a evolução das condições de acesso à alimentação. A HFSSM dos Estados Unidos foi precursora no desenvolvimento de escalas que avaliam o acesso a alimentos com base em recursos financeiros, servindo de inspiração para a *Escala Brasileira de Insegurança Alimentar* (EBIA), adaptada ao contexto do país a partir de 2003 ^8^ ^,^ ^9^ ^,^ ^10^.

Desde então, as propriedades psicométricas da EBIA têm sido amplamente avaliadas, mostrando alta consistência interna e validade ^11^. Sua unidimensionalidade foi corroborada ^12^ e versões de 14 itens têm sido as mais utilizadas em inquéritos nacionais. Os itens são organizados em relação ao nível de gravidade da insegurança alimentar, com distinções para domicílios com apenas adultos e aqueles que também têm crianças e adolescentes ^9^ ^,^ ^10^. A EBIA tem propriedades adequadas, com itens independentes que mapeiam de forma eficaz a crescente intensidade de insegurança alimentar ^10^ ^,^ ^13^. Além disso, é efetiva na classificação de domicílios em quatro categorias: segurança alimentar e insegurança alimentar leve, moderada e grave ^14^ ^,^ ^15^. Estudos adicionais confirmaram a adequação dos pontos de corte que delimitam essas categorias ^16^. Além disso, a validade externa da escala é confirmada por evidências baseadas em fatores como renda, escolaridade e ingestão alimentar ^9^ ^,^ ^10^ ^,^ ^17^.

Contudo, apesar das evidências que apontam excelentes propriedades de estrutura interna (configural, métrica e escalar) e de validade externa da EBIA, uma questão importante e ainda pouco explorada diz respeito à sua invariância de mensuração. A invariância refere-se à capacidade de um instrumento medir de forma consistente o mesmo construto em diferentes grupos populacionais, garantindo que as diferenças observadas entre eles reflitam variações reais no construto, e não resultantes de problemas no funcionamento da escala ^18^ ^,^ ^19^ ^,^ ^20^.

Visando a preencher essa lacuna, recentemente Cezimbra ^21^ examinou invariância da EBIA em grupos populacionais conforme sexo, cor ou raça e escolaridade, considerando a composição familiar do domicílio. Os autores observaram que a EBIA é capaz de produzir estimativas comparáveis entre esses segmentos populacionais. No entanto, ainda existem outros recortes de invariância que precisam ser explorados, como aqueles relacionados a fatores territoriais. É fundamental questionar se a presunção de que a EBIA mede de forma equivalente a insegurança alimentar em todos os territórios se sustenta, e se as comparações refletem desigualdades que derivam da forma como a escala opera nesses diferentes contextos, ou se são factuais, refletindo verdadeiras diferenças nas estimativas de insegurança alimentar. Esse questionamento se torna ainda mais relevante considerando que, desde 2004, a EBIA tem sido usada nos inquéritos brasileiros de grande porte, como a PNAD e a *Pesquisa de Orçamentos Familiares* (POF). Convém salientar que essas estimativas desagregadas por regiões e Unidades da Federação (UF), por exemplo, se apoiam na suposição ainda não formalmente testada de que a EBIA é invariante em todos os segmentos e, assim, que as comparações territoriais divulgadas ao longo dos anos sobre insegurança alimentar seriam, também, válidas por extensão.

É necessário, portanto, investigar se esses achados realmente merecem endosso, o que torna essencial obter mais informações sobre a sustentabilidade da equivalência de funcionamento territorial da EBIA. A fome no Brasil segue fortemente condicionada por determinantes estruturais: desigualdades socioeconômicas e territoriais, além de efeitos recorrentes de crises políticas e econômicas ^22^. Esses determinantes moldam, de forma desigual, o acesso aos alimentos e produzem padrões territoriais de vulnerabilidade que não podem ser compreendidos sem instrumentos sensíveis às diferenças regionais. Assim, a validade psicométrica da EBIA, incluindo a sua comparabilidade entre contextos, torna-se ainda mais crucial: sem assegurar que a escala mensura o mesmo construto em diferentes territórios, corre-se o risco de interpretar desigualdades estruturais como artefatos de mensuração, comprometendo diagnósticos, monitoramento e o planejamento de políticas públicas de enfrentamento da insegurança alimentar. Nesse sentido, e estendendo os achados encontrados em Cezimbra ^21^, o objetivo deste estudo é avaliar e responder à seguinte pergunta de pesquisa: “A EBIA mede a insegurança alimentar de forma equivalente em diferentes contextos territoriais do Brasil - situação urbana e rural, regiões e estados?”.

## Métodos

### Amostra e participantes

Os dados são provenientes da PNAD de 2013, uma pesquisa de representatividade nacional realizada pelo Instituto Brasileiro de Geografia e Estatística (IBGE), cujo objetivo foi fornecer informações para o monitoramento do desenvolvimento socioeconômico do Brasil. O inquérito utilizou uma amostra probabilística por conglomerados em três estágios de seleção, envolvendo as seguintes unidades: municípios, setores censitários e domicílios ^23^ ^,^ ^24^. A amostra final compreendeu 116.543 domicílios. Informações mais detalhadas sobre o processo de amostragem podem ser encontradas nos documentos oficiais do IBGE referentes à PNAD ^23^ ^,^ ^24^. Para o nosso estudo, a amostra completa foi dividida em três partes com a finalidade de avaliar a consistência dos resultados (mais detalhes sobre a divisão são apresentados na subseção *Análise de Dados*).

### Ferramentas e procedimentos de medição

Seguindo as classificações do IBGE para as localizações geográficas dos domicílios ^23^, a amostra foi analisada com base na situação urbana ou rural; nas macrorregiões (Norte, Nordeste, Sudeste, Sul e Centro-oeste); e nos 26 estados, além do Distrito Federal. No presente trabalho, essas três estratificações territoriais são denominadas “situação do domicílio”, “regiões brasileiras” e “UF”, respectivamente.

As análises de invariância focalizaram apenas os oito itens gerais da EBIA, conforme a escala proposta por Segall-Corrêa et al. ^10^. Segundo Interlenghi et al. ^16^, essa versão reduzida pode ser utilizada mesmo em famílias com crianças e adolescentes. Tais itens abordam as seguintes questões: preocupação de que os alimentos acabem antes de poderem comprar ou receber mais comida (i1); se os alimentos acabaram antes que os moradores tivessem dinheiro para comprar mais comida (i2); se os moradores ficaram sem dinheiro para ter uma alimentação saudável e variada (i3); se moradores comeram apenas alguns alimentos porque o dinheiro acabou (i4); se (adulto) deixou de fazer uma refeição porque não havia dinheiro para comprar comida (i5); se (adulto) alguma vez comeu menos do que devia porque não havia dinheiro para comprar comida (i6); se alguma vez (adulto) sentiu fome, mas não comeu, porque não havia dinheiro para comprar comida (i7); e se, alguma vez (adulto), fez apenas uma refeição ao dia ou ficou um dia inteiro sem comer porque não havia dinheiro para comprar comida (i8). A versão completa da escala está disponível em Segall-Corrêa et al. ^10^. Sua aplicação foi realizada por equipes de entrevistadores treinados, durante visitas domiciliares, utilizando *personal digital assistants* (ver detalhes em IBGE ^23^ ^,^ ^24^). Embora a PNAD 2013 disponha da versão ampliada da EBIA, de 14 itens, priorizamos a utilização da versão reduzida devido à evidência de que os itens comuns a todos os domicílios apresentam desempenho psicométrico consistente e alta concordância com a versão completa, inclusive em domicílios com crianças e adolescentes. A literatura também indica que a versão de oito itens mantém propriedades escalares adequadas e pontos de corte estáveis ^13^ ^,^ ^16^.

### Análise de dados

O particionamento da amostra foi realizado usando-se o programa *splitsample* do Stata (v. 16, https://www.stata.com). Para criar um cenário emulando três investigações simultâneas, utilizou-se a opção de balanceamento com base nas variáveis indicadoras de unidades primárias de amostragem e dos pesos amostrais domiciliares. As partições resultaram em tamanhos de amostra de 38.770, 39.003 e 38.770 domicílios, com pesos amostrais expandidos para a população total, conforme esperado.

As análises envolveram duas etapas. A primeira consistiu em uma inspeção preliminar da EBIA com a amostra total, com o objetivo de avaliar a estrutura dimensional da escala e verificar a presença de possíveis correlações residuais entre itens - aspecto importante para as análises de invariância. Inicialmente, implementou-se uma análise paralela de Horn, utilizando-se a análise de componentes principais (ACP) para verificar quantos fatores poderiam ser mantidos, considerando os autovalores ajustados superiores a 1,0 ^25^ ^,^ ^26^. Implementou-se uma análise fatorial confirmatória (AFC) unidimensional, uma vez encontrado apenas um autovalor acima desse limite. Além de reavaliar a dimensionalidade da escala, a AFC também objetivou identificar possíveis violações da independência condicional entre pares de itens. Para isso, focalizou-se nos indicadores de modificação (IM; multiplicador de Lagrange univariado) e respectivos indicadores de mudanças esperadas de parâmetros (MEP), que auxiliam na detecção de correlações residuais ^27^ ^,^ ^28^. Nessa busca, inicialmente direcionamos nossa atenção para IM excepcionalmente altos e MEP que sugerissem a possível presença de correlações residuais padronizadas superiores a 0,30. Qualquer correlação residual que excedesse esse limite seria, então, estimada livremente em um novo modelo, por indicar uma possível violação de independência condicional entre os itens ^29^.

A segunda etapa envolveu a análise de invariância usando-se o método de Alinhamento ^30^ ^,^ ^31^, aplicada separadamente nas três partições da amostra. O método de alinhamento é uma técnica usada para tratar a invariância ao comparar médias fatoriais entre grupos, mesmo quando há indícios de que alguns parâmetros do modelo (cargas ou limiares) possam variar. A técnica busca identificar quais parâmetros se mantêm estáveis entre os grupos e quais apresentam diferenças substanciais, preservando o máximo de parâmetros invariantes. Assim, o método produz um modelo próximo à invariância configural, mas destacando apenas os parâmetros cuja variação é grande o suficiente para indicar violação de invariância. Aceita-se um modelo com invariância parcial quando, pelo menos, 80% dos parâmetros são invariantes ^19^ ^,^ ^31^ ^,^ ^32^.

As análises usaram modelos *probit* com matrizes de entrada baseadas em correlações tetracóricas e empregaram o estimador de mínimos quadrados ponderados diagonalmente (ou *weighted least squares mean and varianca adjusted*; WLSMV, acrônimo em inglês). Esse estimador é apropriado para lidar com variáveis dicotômicas, como os itens da EBIA ^33^. Os ajustes dos modelos foram verificados por meio do erro quadrático médio de aproximação (*root mean square error of approximation*; RMSEA, acrônimo em inglês), do índice de ajuste comparativo (CFI) e do índice de Tucker-Lewis (TLI) ^27^. Valores de RMSEA < 0,06 indicam um bom ajuste do modelo, já os valores > 0,10 apontam para um ajuste inadequado, levando à rejeição do modelo. Os índices de CFI e TLI variam de 0 a 1, sendo que valores > 0,95 são considerados indicativos de um ajuste satisfatório ^27^ ^,^ ^34^.

Utilizou-se a configuração de alinhamento fixo nas análises de invariância focalizando a situação do domicílio, recomendada quando se compara poucos grupos: define-se um grupo de referência cuja média fatorial é fixada em zero, e os demais são comparados a ele. A configuração livre, em que as médias fatoriais de todos os grupos são estimadas livremente, foi utilizada para as análises de invariância entre regiões e estados brasileiros ^31^. Optou-se pela parametrização *theta*, uma vez que a parametrização alternativa, delta, fornece resultados intercambiáveis nas análises. Para identificar parâmetros invariantes e não invariantes, adotou-se o ponto de corte de p > 0,001 nas comparações de cargas e limiares entres os grupos.

Além do processamento das partições, o pacote estatístico Stata (https://www.stata.com) foi utilizado para a organização do banco de dados, as análises de distribuição e a análise paralela de Horn. As análises fatoriais e de invariância pelo método de Alinhamento foram conduzidas no Mplus (versão 8.8; https://www.statmodel.com/). Todas as análises levaram em conta os procedimentos complexos de amostragem.

## Resultados

A Tabela 1 apresenta as características demográficas dos domicílios analisados. A maior parte deles (85,7%) estava localizada em áreas urbanas. Cerca de 43% pertenciam à Região Sudeste, e apenas 7,3% estavam na Região Norte. Aproximadamente 22% dos domicílios estavam situados no Estado de São Paulo.

**Tabela 1:** Distribuição da amostra de acordo com situação urbana/rural, regiões e Unidades da Federação (UF) do domicílio nas três partições e amostra geral. *Pesquisa Nacional por Amostra de Domicílios* (PNAD) *Contínua*, Brasil, 2013*.*

Características	Partição 1 (n = 38.770)		Partição 2 (n = 39.003)		Partição 3 (n = 38.770)		Amostra total (n = 116.543)	
	n	% *	n	% *	n	% *	n	% *
Situação urbana/rural								
Urbana	33.438	85,99	33.588	85,73	33.345	85,59	100.371	85,77
Rural	5.332	14,01	5.415	14,26	5.425	14,41	16.172	14,23
Regiões brasileiras								
Norte	5.570	7,31	5.626	7,33	5.560	7,30	16.756	7,31
Nordeste	10.784	26,24	10.811	26,12	10.774	26,21	32.369	26,19
Sudeste	11.759	43,44	11.880	43,66	11.759	43,44	35.398	43,52
Sul	6.517	15,35	6.517	15,22	6.537	15,38	19.571	15,32
Centro-oeste	4.140	7,66	4.169	7,67	4.140	7,66	12.449	7,66
UF								
Rondônia	764	0,86	763	0,85	749	0,84	2.276	0,85
Acre	420	0,34	420	0,33	420	0,34	1.260	0,34
Amazonas	1.101	1,49	1.096	1,47	1.106	1,49	3.303	10,48
Roraima	275	0,22	281	0,23	275	0,22	831	0,23
Pará	2.089	3,40	2.149	3,45	2.089	3,40	6.327	3,42
Amapá	259	0,30	246	0,29	259	0,30	764	0,30
Tocantins	662	0,70	671	0,70	662	0,70	1.995	0,70
Maranhão	834	2,82	848	2,85	834	2,82	2.516	2,83
Piauí	545	1,43	543	1,42	545	1,43	1.633	1,42
Ceará	1.926	4,09	1.914	4,05	1.940	4,11	5.780	4,08
Rio Grande do Norte	516	1,58	523	1,60	511	1,57	1.550	1,58
Paraíba	640	1,87	637	1,85	640	1,87	1.917	1,86
Pernambuco	2.190	4,47	2.201	4,48	2.185	4,47	6.576	4,47
Alagoas	534	1,49	532	1,47	534	1,49	1.600	1,48
Sergipe	629	1,05	657	1,09	629	1,05	1.915	1,06
Bahia	2.970	7,44	2.956	7,32	2.956	7,42	8.882	7,39
Minas Gerais	3.636	10,43	3.678	10,48	3.636	10,43	10.950	10,45
Espírito Santo	762	2,00	769	2,01	762	2,00	2.293	2,01
Rio de Janeiro	3.020	8,87	3.022	8,82	3.020	8,87	9.062	8,85
São Paulo	4.341	22,14	4.411	22,35	4.341	22,14	13.093	22,21
Paraná	2.154	5,74	2.147	5,68	2.159	5,75	6.460	5,72
Santa Catarina	1.130	3,50	1.127	3,47	1.130	3,50	3.387	3,49
Rio Grande do Sul	3.233	6,11	3.243	6,07	3.248	6,12	9.724	6,10
Mato Grosso do Sul	702	1,33	710	1,33	702	1,33	2.114	1,33
Mato Grosso	805	1,65	802	1,64	805	1,65	2.412	1,65
Goiás	1.627	3,29	1.653	3,32	1.627	3,29	4.907	3,30
Distrito Federal	1.006	1,39	1.004	1,37	1.006	1,39	3.016	1,38

* Frequência relativa, ajustada pelos pesos amostrais.

Os diagnósticos iniciais com base nos dados agregados indicaram uma possível correlação residual entre os itens i5 e i8 (IM = 151.779), levando a um valor de 0,359 quando livremente estimado. Todos os modelos basilares para cada estrato de cada subgrupo mostraram índices RMSEA, CFI e TLI sugestivos de ajuste adequado (Material Suplementar; https://cadernos.ensp.fiocruz.br/static//arquivo/suppl-e00115125_5547.pdf).

Reiterando, a invariância pelo método de Alinhamento foi testada para todos os grupos, em cada partição. Não houve violação de invariância de cargas e limiares dos itens ao se comparar as áreas urbana e rural. Por sua vez, nas partições 1 e 3, identificou-se cargas não invariantes ao se contrastar as regiões Sul e Nordeste no item 1, e Sudeste e Nordeste no item 2. Uma violação se apresentou no limiar do item 4 de três pares confrontados: regiões Sudeste e Norte, Sul e Norte, e Centro-oeste e Norte. Também foi detectado um limiar não invariante no item 8 envolvendo as regiões Norte e Nordeste nas partições 1 e 2. Focalizando as UF, em todas as partições houve violações de invariância de limiar relativa ao item 4 envolvendo a comparação do estado do Amazonas com Bahia, Paraná e Goiás.

Observando todas as possibilidades, encontrou-se 0,6% de violações de invariância. O cálculo considerou as três partições, os oito itens da escala, os dois parâmetros por contraste (carga e limiar) e os três agrupamentos principais envolvendo as duas categorias de situação urbana e rural, as cinco regiões e os 26 estados brasileiros e Distrito Federal. De todas as 17.376 possibilidades, somente 103 mostraram alguma violação. Vinte e uma foram encontradas nas análises das regiões e 82 nas UF; nenhuma foi detectada na situação urbana/rural.

Os resultados completos para situação urbana e rural, assim como regiões brasileiras em cada partição, estão no Material Suplementar (https://cadernos.ensp.fiocruz.br/static//arquivo/suppl-e00115125_5547.pdf). Para evitar uma extensiva apresentação de resultados, mostramos apenas os valores de cargas e limiares dos itens que apresentaram diferenças significativas no segmento estados brasileiros (Material Suplementar; https://cadernos.ensp.fiocruz.br/static//arquivo/suppl-e00115125_5547.pdf). A síntese dos resultados da análise de invariância pelo método de Alinhamento para os grupos e partições analisados está no Quadro 1, no qual também são destacados apenas os itens significativamente diferentes entre as UF.

**Quadro 1:** Síntese da análise de invariância da *Escala Brasileira de Insegurança Alimentar* (versão reduzida, de 8 itens) pelo método de Alinhamento, segundo situação urbana/rural, regiões e Unidades da Federação (UF) do domicílio, por partição. *Pesquisa Nacional por Amostra de Domicílios* (PNAD) *Contínua*, Brasil, 2013.

Itens	i1			i2			i3			i4			i5			i6			i7			i8		
Partições	1	2	3	1	2	3	1	2	3	1	2	3	1	2	3	1	2	3	1	2	3	1	2	3
Situação urbana/rural																								
Rural x urbana																								
Regiões brasileiras																								
Nordeste x Norte			●																					
Sudeste x Norte																								
Sudeste x Nordeste			●	●		●																		
Sul x Norte				●																				
Sul x Nordeste	●		●	●																				
Sul x Sudeste																								
Centro-oeste x Norte																								
Centro-oeste x Nordeste				●																				
Centro-oeste x Sudeste																								
Centro-oeste x Sul																								
UF																								
RR x AM																								
PA x AM																								
TO x AM																								
MA x AM																								
MA x TO																								
PI x AM																								
PI x AP																								
PI x MA																								
CE x AM																								
CE x TO																								
RN x AM																								
PB x AM																		●						
PB x PA																		●						
PB x MA																		●						
PB x CE																		●						
PE x AM																								
PE x TO																								
PE x PB																		●						
SE x AM																								
SE x PA																								
BA x AM																								
BA x RR																								
BA x MA																								
BA x PB																		●						
MG x AM																								
MG x PA																								
MG x PB																		●						
MG x BA																								
ES x TO																								
ES x SE																								
RJ x AM																								
RJ x PB																		●						
SP x AM																								
SP x MA																								
SP x PB																		●						
PR x AM																								
SC x AM																								
SC x TO																								
SC x MA																								
SC x PI																								
SC x CE																								
SC x PB																								
SC x BA																								
RS x AM																								
RS x MA																								
MS x AM																								
MT x AM																								
MT x PI																								
GO x AM																								
GO x RR																								
GO x PI																								
GO x PE																								
GO x SE																								
GO x ES																								
DF x AM																								

AM: Amazonas; AP: Amapá; BA: Bahia; CE: Ceará; DF: Distrito Federal; ES: Espírito Santo; GO: Goiás; MA: Maranhão; MG: Minas Gerais; MS: Mato Grosso do Sul; MT: Mato Grosso; PA: Pará; PB: Paraíba; PE: Pernambuco; PI: Piauí; PR: Paraná; RJ: Rio de Janeiro; RN: Rio Grande do Norte; RR: Roraima; RS: Rio Grande do Sul; SC: Santa Catarina; SE: Sergipe; SP: São Paulo; TO: Tocantins.

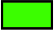
 Invariância (carga e limiar);

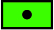
 Violação de invariância de carga;

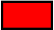
 Violação de invariância de limiar;

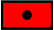
 Violação de invariância de carga e limiar.

## Discussão

Conforme exposto anteriormente, a EBIA é há bastante tempo utilizada como ferramenta para medir a insegurança alimentar domiciliar em inquéritos nacionais. Estudos prévios já atestaram suas excelentes propriedades psicométricas, reforçando sua efetividade na aferição desse fenômeno. Focalizando a invariância, Hackett et al. ^35^ analisaram a EBIA quanto ao sexo do respondente, e Cezimbra ^21^ expandiu essa análise para grupos cor/raça e escolaridade também, levando em conta a composição familiar do domicílio. Contudo, ainda faltavam investigações centradas na invariância segundo diferentes segmentos territoriais, como áreas urbanas e rurais, regiões e UF. O presente estudo procurou preencher essa importante lacuna, demonstrando que a versão de oito itens da EBIA é invariante aqui também e, assim, avalizando comparações válidas e confiáveis entre os segmentos.

A EBIA é essencial para identificar famílias vulneráveis à insegurança alimentar. Ao longo dos anos, os dados gerados por essa escala têm sido amplamente aceitos como representações válidas e confiáveis da realidade da insegurança alimentar em diferentes segmentos populacionais. Nossos resultados sugerem que as comparações entre áreas urbanas e rurais, bem como entre regiões e estados brasileiros, são confiáveis e não estão sujeitas a vieses de mensuração.

Ainda assim, algumas violações de cargas, limiares ou ambas conjuntamente foram detectadas em alguma partição, agrupamento e item. O item 4 (se moradores comeram apenas alguns alimentos porque o dinheiro acabou) apresentou diferenças consideráveis entre algumas regiões e em comparações envolvendo UF, principalmente em relação ao Estado do Amazonas. O item 3, que questiona “se os moradores ficaram sem dinheiro para ter uma alimentação saudável e variada”, também apresentou diferenças claras em algumas comparações envolvendo essa mesma UF. Esse perfil específico referente ao Amazonas está apresentado no Material Suplementar (https://cadernos.ensp.fiocruz.br/static//arquivo/suppl-e00115125_5547.pdf). Tais divergências sugerem que há particularidades regionais que afetam a maneira como esses itens da escala são compreendidos. Sobretudo nesse estado, isso pode ser reflexo das condições socioculturais específicas modificando a percepção dessa população quanto à ideia de “dinheiro acabar” ou o que constitui uma alimentação “saudável e variada”. A sazonalidade dos rios afeta diretamente as atividades de pesca, produção e circulação de alimentos, impactando, consequentemente, tanto a disponibilidade de alimentos regionais quanto os recursos financeiros da população e acesso a alimentos diversificados ^36^ ^,^ ^37^. A combinação entre vulnerabilidade econômica, limitações dos meios de transporte, custos elevados, menor oferta de alimentos regionais e riscos climáticos configuram barreiras concretas ao abastecimento alimentar na região ^36^ ^,^ ^37^ ^,^ ^38^. Assim, a interpretação dos itens da EBIA pode variar não apenas por fatores culturais, mas também pelas desigualdades territoriais e materiais que moldam o cotidiano alimentar. Incorporar essas dimensões ajuda a compreender por que certas violações de invariância emergem em contextos específicos e reforça a importância de análises sensíveis aos determinantes estruturais das desigualdades regionais.

A despeito desses achados, a magnitude das violações requer perspectiva, já que apenas 0,6% dos contrastes de carga ou limiares indicou não-invariância. Essa evidência nos permitiria considerar a EBIA como “quase” invariante por todos os fins práticos, ainda que formalmente a situação deveria ser rotulada como de invariância parcial ^19^ ^,^ ^30^ ^,^ ^32^. Claramente, o desempenho da escala é amplamente positivo, reforçando sua validade e confiabilidade para comparações entre os contextos analisados, especialmente quando situado no cenário atual da insegurança alimentar no país. Apesar da redução das prevalências de insegurança alimentar entre 2022 e 2024 ^4^ ^,^ ^22^ e da recente saída do Brasil do Mapa da Fome pela FAO, persistem disparidades profundas, sobretudo no Norte e no Nordeste, onde condições estruturais continuam a limitar o acesso regular a alimentos ^22^. Assim, ao demonstrar que a EBIA mantém desempenho psicométrico consistente entre segmentos territoriais, nossos achados fortalecem a confiabilidade de indicadores fundamentais para monitorar desigualdades persistentes e orientar políticas de combate à fome em contextos de grandes oscilações sociais e econômicas.

O presente estudo tem algumas limitações a serem consideradas. Os dados utilizados são de 2013, o que significa que os resultados podem não se manter ao longo do tempo. Embora o uso da PNAD 2013 não comprometa a interpretação dos achados quanto às propriedades psicométricas da EBIA, não é possível assegurar que a invariância se mantenha diante de transformações socioeconômicas. Dessa forma, recomenda-se que estudos futuros examinem a invariância longitudinalmente para garantir a consistência dos parâmetros da escala ao longo dos anos. Embora a versão de oito itens da EBIA seja validada como confiável para famílias com crianças e adolescentes ^16^, é fundamental, em estudos futuros, também avaliar a invariância da versão completa, de 14 itens, a fim de garantir que as medições de insegurança alimentar sejam consistentes entre diferentes grupos populacionais, assegurando sua validade e comparabilidade - ainda que grandes diferenças possam não ser encontradas em relação aos resultados observados em nosso estudo.

Além disso, apesar da investigação em diferentes localizações dos domicílios, é essencial que futuras pesquisas explorem aspectos adicionais. A EBIA pode não se mostrar invariante, e a estimativa de insegurança alimentar pode não ser comparável em outros grupos e contextos que não foram analisados em nosso trabalho. Não analisamos a invariância considerando a intersecção de grupos como, por exemplo, entre diferentes grupos demográficos, como domicílios urbanos e rurais em diversas regiões ou diferentes estratos demográficos e sociais. Portanto, futuros trabalhos devem produzir evidências acerca da invariância do instrumento em relação a essas e outras intersecções mencionadas. O estudo apresenta, por outro lado, pontos positivos, evidenciados pela sua abordagem abrangente e metodologicamente rigorosa. Isso inclui, por exemplo, a replicação em três partições da amostra para a análise da invariância, fortalecendo a confiabilidade dos nossos achados, assegurando que sejam consistentes em diferentes grupos populacionais.

## Conclusão

Em suma, nossos resultados afirmam a validade da versão de oito itens da EBIA como ferramenta eficaz para mensurar a insegurança alimentar em diversos segmentos territoriais. Contudo, a observação de violações de invariância em alguns itens, principalmente no Estado do Amazonas, sugere a necessidade de mais investigações que considerem especificidades regionais. Esses achados têm implicações importantes para o monitoramento da fome e para o desenho de políticas públicas de segurança alimentar e nutricional no Brasil: ao demonstrar que a EBIA opera de maneira equivalente entre diferentes segmentos territoriais, fortalecemos a credibilidade dos indicadores utilizados em inquéritos nacionais, que orientam a identificação de populações mais vulneráveis, a alocação de recursos e expansão de programas sociais. Ao mesmo tempo, as diferenças regionais detectadas reforçam a importância de acompanhar potenciais variações nas propriedades de mensuração da escala, assegurando que a EBIA continue fornecendo medidas importantes para apoiar o planejamento, o monitoramento e a avaliação de políticas públicas voltadas ao enfrentamento da fome.

## Data Availability

Os dados de pesquisa estão disponíveis mediante solicitação à autora de correspondência.

## Material Suplementar

Material Suplementar
