# Supplementary material for: Invariance of the Brazilian Food Insecurity Scale: analysis of demographic groups based on the Brazilian National Household Sample Survey
Source: Cad Saude Publica. 2026 Jul 24;42:e00115125. [Article in Portuguese] doi: 10.1590/0102-311XPT115125 (PMC13411253; doi:10.1590/0102-311XPT115125)
Supplement: Material Suplementar [file 1678-4464-csp-42-PT115125-s.pdf]

Material suplementar

Tabela S1 Índices de ajustes da Análise Fatorial Confirmatória na *Escala Brasileira de Insegurança Alimentar* (versão reduzida, 8 itens), de acordo com situação urbana e rural, regiões e Unidades da Federação (UF) dos domicílios, por partição. PNAD, 2013

	χ2	df	χ2diff	Δdf	RMSEA (90%IC)	CFit	SRMR	CFI	TLI
					**Partição 1**				
**Situação urbana/rural**									
**Modelos basilares**									
Urbano	997.887*	19	-	-	0.039 (0.037-0.041)	1.000	0.031	0.999	0.998
Rural	312.795*	19	-	-	0.054 (0.049-0.059)	1.008	0.039	0.997	0.996
**Regiões brasileiras**									
**Modelos basilares**									
Norte	367.496*	19	-	-	0.057 (0.052-0.063)	0.008	0.034	0.998	0.997
Nordeste	694.378*	19	-	-	0.057 (0.054-0.061)	0.000	0.045	0.997	0.995
Sudeste	212.160*	19	-	-	0.029 (0.026-0.033)	1.000	0.026	0.999	0.999
Sul	112.470*	19	-	-	0.027 (0.023-0.032)	1.000	0.025	0.999	0.999
Centro-oeste	119.638*	19	-	-	0.036 (0.030-0.042)	1.000	0.034	0.999	0.998
**UF**									
**Modelos basilares**									
Rondônia	40.913*	19	-	-	0.039 (0.022-0.055)	0.860	0.049	0.998	0.998
Acre	25.169*	19	-	-	0.028 (0.000-0.054)	0.911	0.015	1.000	1.000
Amazonas	117.125*	19	-	-	0.068 (0.057-0.081)	0.005	0.041	0.996	0.995
Roraima	26.781*	19	-	-	0.039 (0.000-0.070)	0.689	0.069	0.998	0.997
Pará	149.588*	19	-	-	0.057 (0.049-0.066)	0.073	0.035	0.998	0.997
Amapá	18.132*	19	-	-	0.000 (0.000-0.052)	0.940	0.031	1.000	1.000
Tocantins	57.698*	19	-	-	0.055 (0.039-0.072)	0.269	0.067	0.998	0.996
Maranhão	96.589*	19	-	-	0.070 (0.056-0.084)	0.008	0.058	0.993	0.990
Piauí	67.356*	19	-	-	0.068 (0.051-0.086)	0.041	0.070	0.994	0.991
Ceará	109.784*	19	-	-	0.050 (0.041-0.059)	0.495	0.043	0.997	0.996
Rio Grande do Norte	32.875*	19	-	-	0.038 (0.013-0.059)	0.817	0.039	0.999	0.999
Paraíba	43.204*	19	-	-	0.045 (0.027-0.062)	0.668	0.046	0.999	0.998
Pernambuco	87.956*	19	-	-	0.041 (0.032-0.049)	0.959	0.046	0.998	0.997
Alagoas	62.417*	19	-	-	0.065 (0.048-0.084)	0.073	0.065	0.997	0.996
Sergipe	24.755*	19	-	-	0.022 (0.000-0.044)	0.987	0.049	0.999	0.999
Bahia	219.035*	19	-	-	0.060 (0.053-0.067)	0.012	0.048	0.997	0.995
Minas Gerais	83.764*	19	-	-	0.031 (0.024-0.037)	1.000	0.038	0.998	0.998
Espírito Santo	34.085*	19	-	-	0.032 (0.013-0.049)	0.955	0.019	1.000	0.999
Rio de Janeiro	57.010*	19	-	-	0.026 (0.018-0.034)	1.000	0.018	1.000	0.999
São Paulo	84.629*	19	-	-	0.028 (0.022-0.034)	1.000	0.026	0.999	0.999
Paraná	46.625*	19	-	-	0.026 (0.017-0.036)	1.000	0.028	0.999	0.999
Santa Catarina	29.118*	19	-	-	0.022 (0.000-0.037)	1.000	0.025	0.999	0.999
Rio Grande do Sul	48.429*	19	-	-	0.022 (0.014-0.030)	1.000	0.025	0.999	0.999
Mato Grosso do Sul	23.099*	19	-	-	0.018 (0.000-0.039)	0.996	0.022	1.000	0.999

Mato Grosso	39.401*	19	-	-	0.037 (0.020-0.053)	0.912	0.048	0.998	0.997
Goiás	72.507*	19	-	-	0.042 (0.032-0.052)	0.906	0.046	0.998	0.998
Distrito Federal	39.101*	19	-	-	0.032 (0.018-0.047)	0.979	0.029	0.999	0.999
					**Partição 2**				
**Situação urbana/rural**									
**Modelos basilares**									
Urbano	1085.449*	19	-	-	0.041 (0.039-0.043)	1.000	0.029	0.998	0.998
Rural	294.303*	19	-	-	0.052 (0.047-0.057)	0.283	0.036	0.998	0.997
**Regiões brasileiras**									
**Modelos basilares**									
Norte	282.370*	19	-	-	0.050 (0.045-0.055)	0.535	0.030	0.998	0.996
Nordeste	751.809*	19	-	-	0.060 (0.056-0.063)	0.000	0.046	0.996	0.994
Sudeste	218.561*	19	-	-	0.030 (0.026-0.033)	1.000	0.024	0.999	0.999
Sul	144.744*	19	-	-	0.032 (0.027-0.037)	1.000	0.025	0.999	0.999
Centro-Oeste	97.636*	19	-	-	0.032 (0.025-0.038)	1.000	0.026	0.999	0.999
**UF**									
**Modelos basilares**									
Rondônia	30.232*	19	-	-	0.028 (0.002-0.046)	0.982	0.033	0.999	0.999
Acre	28.646*	19	-	-	0.035 (0.000-0.059)	0.829	0.030	1.000	0.999
Amazonas	106.602*	19	-	-	0.065 (0.053-0.077)	0.019	0.042	0.995	0.992
Roraima	19.358*	19	-	-	0.008 (0.000-0.053)	0.931	0.025	1.000	1.000
Pará	110.412*	19	-	-	0.047 (0.039-0.056)	0.681	0.030	0.998	0.998
Amapá	28.686*	19	-	-	0.046 (0.000-0.078)	0.551	0.079	0.998	0.997
Tocantins	32.564*	19	-	-	0.033 (0.011-0.051)	0.937	0.039	0.998	0.997
Maranhão	100.649*	19	-	-	0.071 (0.058-0.085)	0.005	0.060	0.993	0.990
Piauí	70.497*	19	-	-	0.071 (0.053-0.089)	0.025	0.089	0.989	0.984
Ceará	134.031*	19	-	-	0.056 (0.047-0.065)	0.118	0.045	0.997	0.996
Rio Grande do Norte	33.039*	19	-	-	0.038 (0.014-0.059)	0.820	0.043	0.999	0.998
Paraíba	56.759*	19	-	-	0.056 (0.039-0.073)	0.6260	0.072	0.997	0.995
Pernambuco	74.031*	19	-	-	0.036 (0.028-0.045)	0.995	0.035	0.998	0.998
Alagoas	31.219*	19	-	-	0.035 (0.008-0.056)	0.872	0.038	0.999	0.999
Sergipe	41.292*	19	-	-	0.042 (0.024-0.060)	0.746	0.066	0.998	0.997
Bahia	239.819*	19	-	-	0.063 (0.056-0.070)	0.001	0.045	0.997	0.995
Minas Gerais	94.198*	19	-	-	0.033 (0.026-0.040)	1.000	0.031	0.999	0.998
Espírito Santo	37.071*	19	-	-	0.035 (0.018-0.052)	0.925	0.039	1.000	0.999
Rio de Janeiro	44.354*	19	-	-	0.021 (0.013-0.029)	1.000	0.024	1.000	0.999
São Paulo	73.631*	19	-	-	0.026 (0.020-0.032)	1.000	0.019	0.999	0.999
Paraná	61.404*	19	-	-	0.032 (0.023-0.041)	0.999	0.029	0.999	0.999
Santa Catarina	32.596*	19	-	-	0.025 (0.009-0.040)	0.999	0.031	0.999	0.999
Rio Grande do Sul	61.773*	19	-	-	0.026 (0.019-0.034)	1.000	0.024	0.999	0.999
Mato Grosso do Sul	39.106*	19	-	-	0.039 (0.021-0.056)	0.853	0.046	0.999	0.999
Mato Grosso	29.951*	19	-	-	0.027 (0.000-0.044)	0.988	0.034	0.999	0.999
Goiás	36.360*	19	-	-	0.024 (0.011-0.035)	1.000	0.024	1.000	0.999
Distrito Federal	29.070*	19	-	-	0.023 (0.000-0.039)	0.999	0.032	1.000	0.999
					**Partição 3**				
**Situação urbana/rural**									

**Modelos basilares**									
Urbano	989.944*	19	-	-	0.039 (0.037-0.041)	1.000	0.030	0.999	0.998
Rural	345.621*	19	-	-	0.056 (0.051-0.062)	0.022	0.043	0.997	0.996
**Regiões brasileiras**									
**Modelos basilares**									
Norte	360.416*	19	-	-	0.057 (0.052-0.062)	0.013	0.034	0.998	0.997
Nordeste	679.446*	19	-	-	0.057 (0.053-0.061)	0.001	0.046	0.997	0.995
Sudeste	177.741*	19	-	-	0.027 (0.023-0.030)	1.000	0.022	0.999	0.999
Sul	174.280*	19	-	-	0.035 (0.031-0.040)	1.000	0.034	0.998	0.998
Centro-oeste	119.040*	19	-	-	0.036 (0.030-0.042)	1.000	0.033	0.999	0.998
**UF**									
**Modelos basilares**									
Rondônia	33.345*	19	-	-	0.032 (0.012-0.049)	0.958	0.040	0.999	0.999
Acre	18.107*	19	-	-	0.000 (0.000-0.041)	0.989	0.012	1.000	1.000
Amazonas	118.886*	19	-	-	0.069 (0.057-0.081)	0.004	0.039	0.997	0.995
Roraima	30.433*	19	-	-	0.047 (0.006-0.076)	0.533	0.051	0.998	0.997
Pará	148.314*	19	-	-	0.057 (0.049-0.066)	0.081	0.034	0.998	0.998
Amapá	24.825*	19	-	-	0.034 (0.000-0.068)	0.742	0.055	0.999	0.998
Tocantins	60.575*	19	-	-	0.057 (0.042-0.074)	0.206	0.076	0.996	0.994
Maranhão	74.095*	19	-	-	0.059 (0.045-0.073)	0.136	0.050	0.995	0.993
Piauí	56.665*	19	-	-	0.060 (0.043-0.079)	0.159	0.069	0.996	0.994
Ceará	108.348*	19	-	-	0.049 (0.040-0.058)	0.536	0.050	0.997	0.9965
Rio Grande do Norte	32.875*	19	-	-	0.029 (0.000-0.052)	0.935	0.039	1.000	0.999
Paraíba	77.281*	19	-	-	0.069 (0.054-0.086)	0.023	0.094	0.997	0.996
Pernambuco	76.974*	19	-	-	0.037 (0.029-0.046)	0.991	0.040	0.998	0.997
Alagoas	63.738*	19	-	-	0.066 (0.049-0.085)	0.061	0.061	0.998	0.997
Sergipe	52.511*	19	-	-	0.053 (0.036-0.070)	0.361	0.049	0.998	0.997
Bahia	179.812*	19	-	-	0.054 (0.047-0.061)	0.199	0.045	0.998	0.996
Minas Gerais	59.455*	19	-	-	0.024 (0.017-0.031)	1.000	0.028	0.999	0.999
Espírito Santo	28.772*	19	-	-	0.026 (0.000-0.044)	0.987	0.021	1.000	0.999
Rio de Janeiro	78.034*	19	-	-	0.032 (0.025-0.040)	1.000	0.028	0.999	0.998
São Paulo	64.493*	19	-	-	0.023 (0.017-0.030)	1.000	0.021	1.000	0.999
Paraná	48.019*	19	-	-	0.027 (0.017-0.036)	1.000	0.023	0.999	0.999
Santa Catarina	44.723*	19	-	-	0.035 (0.022-0.048)	0.973	0.042	0.999	0.999
Rio Grande do Sul	99.280*	19	-	-	0.036 (0.029-0.043)	0.999	0.046	0.998	0.997
Mato Grosso do Sul	45.265*	19	-	-	0.044 (0.028-0.061)	0.688	0.035	0.998	0.998
Mato Grosso	28.800*	19	-	-	0.025 (0.000-0.043)	0.991	0.038	0.999	0.999
Goiás	55.499*	19	-	-	0.034 (0.024-0.045)	0.993	0.039	0.999	0.999
Distrito Federal	29.963*	19	-	-	0.024 (0.000-0.040)	0.998	0.029	0.999	0.999

Nota: IC (Intervalo de Confiança de 90%); χ2 (*chi-square statistics*); df (*Degrees of Freedom*); χ2diff (χ2 *difference*); Δdf (diferença entre *Degrees of Freedom* do modelo configural e escalar); RMSEA (*Root Mean Square Error of Approximation*); CFit (*test of close fit* - probabilidade RMSEA ≤ ,05); SRMR (*Standardized Root Mean Square Residual*); CFI (*Comparative Fit Index*) TLI (*Tucker–Lewis Index*). * p<0,001

Tabela S2 Cargas fatoriais não padronizadas do método de Alinhamento na *Escala Brasileira de Insegurança Alimentar* (versão reduzida, de 8 itens) de acordo com situação urbana e rural do domicílio, por partição. PNAD, 2013

Partição 1

Grupos		Cargas fatoriais não padronizadas		Diferença	Erro-padrão da diferença	Valor de p
**ITEM 1**						
**Rural**	**x**	3.107	2.818	0.289	0.198	0.144
**Urbano**						
**R^2**				0.000		
**ITEM 2**						
**Rural**	**x**	3.946	3.229	0.717	0.260	0.006
**Urbano**						
**R^2**				0.000		
**ITEM 3**						
**Rural**	**x**	4.570	4.436	0.134	0.351	0.702
**Urbano**						
**R^2**				0.908		
**ITEM 4**						
**Rural**	**x**	3.688	3.988	-0.300	0.263	0.255
**Urbano**						
**R^2**				0.856		
**ITEM 5**						
**Rural**	**x**	2.995	3.019	-0.024	0.157	0.878
**Urbano**						
**R^2**				0.997		
**ITEM 6**						
**Rural**	**x**	2.976	3.222	-0.246	0.158	0.120
**Urbano**						
**R^2**				0.854		
**ITEM 7**						
**Rural**	**x**	3.634	3.681	-0.046	0.253	0.855
**Urbano**						
**R^2**				0.992		
**ITEM 8**						
**Rural**	**x**	2.758	2.809	-0.051	0.160	0.749
**Urbano**						

Partição 2

Grupos		Cargas fatoriais não padronizadas		Diferença	Erro-padrão da diferença	Valor de p
**ITEM 1**						
**Rural**	**x**	3.242	2.627	0.615	0.228	0.007
**Urbano**						
**R^2**				0.000		
**ITEM 2**						
**Rural**	**x**	3.771	3.180	0.592	0.251	0.018
**Urbano**						
**R^2**				0.000		
**ITEM 3**						
**Rural**	**x**	5.102	4.264	0.838	0.515	0.104
**Urbano**						
**R^2**				0.000		
**ITEM 4**						
**Rural**	**x**	3.641	3.853	-0.213	0.232	0.359
**Urbano**						
**R^2**				0.928		
**ITEM 5**						
**Rural**	**x**	3.002	3.346	-0.344	0.206	0.095
**Urbano**						
**R^2**				0.831		
**ITEM 6**						
**Rural**	**x**	3.471	3.585	-0.114	0.180	0.526
**Urbano**						
**R^2**				0.970		
**ITEM 7**						
**Rural**	**x**	3.772	3.829	-0.057	0.221	0.797
**Urbano**						
**R^2**				0.992		
**ITEM 8**						
**Rural**	**x**	2.643	2.738	-0.095	0.151	0.529
**Urbano**						

Partição 3

Grupos		Cargas fatoriais não padronizadas		Diferença	Erro-padrão da diferença	Valor de p
**ITEM 1**						
**Rural**	**x**	2.684	2.676	0.008	0.135	0.955
**Urbano**						
**R^2**				0.999		
**ITEM 2**						
**Rural**	**x**	3.414	3.400	0.014	0.172	0.934
**Urbano**						
**R^2**				0.998		
**ITEM 3**						
**Rural**	**x**	3.990	4.176	-0.186	0.298	0.534
**Urbano**						
**R^2**				0.903		
**ITEM 4**						
**Rural**	**x**	3.899	3.951	-0.051	0.234	0.826
**Urbano**						
**R^2**				0.987		
**ITEM 5**						
**Rural**	**x**	3.451	3.108	0.343	0.251	0.172
**Urbano**						
**R^2**				0.000		
**ITEM 6**						
**Rural**	**x**	3.388	3.374	0.014	0.173	0.935
**Urbano**						
**R^2**				0.998		
**ITEM 7**						
**Rural**	**x**	4.238	4.034	0.203	0.353	0.565
**Urbano**						
**R^2**				0.077		
**ITEM 8**						
**Rural**	**x**	2.561	2.879	-0.318	0.195	0.103
**Urbano**						

**R^2**	0.984	**R^2**	0.966	**R^2**	0.711

Tabela S3 Limiares do método de Alinhamento na *Escala Brasileira de Insegurança Alimentar* (versão reduzida, de 8 itens) de acordo com situação urbana e rural do domicílio, por partição. PNAD, 2013

Partição 1						Partição 2						Partição 3					
**Grupos**	**Limiares**		**Diferença**	**Erro-padrão da diferença**	**Valor de p**	**Grupos**	**Limiares**		**Diferença**	**Erro-padrão da diferença**	**Valor de p**	**Grupos**	**Limiares**		**Diferença**	**Erro-padrão da diferença**	**Valor de p**
**ITEM 1**																	
**Rural x Urbano**	-0.285	-0.353	0.068	0.055	0.218	**Rural x Urbano**	-0.272	-0.306	0.035	0.048	0.469	**Rural x Urbano**	-1.567	-1.629	0.062	0.094	0.511
**R²**			0.941			**R²**			0.744			**R²**			0.997		
**ITEM 2**																	
**Rural x Urbano**	0.482	0.499	-0.017	0.054	0.753	**Rural x Urbano**	0.490	0.470	0.020	0.044	0.646	**Rural x Urbano**	-1.305	-1.143	-0.162	0.100	0.106
**R²**			0.809			**R²**			0.869			**R²**			0.994		
**ITEM 3**																	
**Rural x Urbano**	0.010	0.208	-0.197	0.078	0.012	**Rural x Urbano**	0.017	0.183	-0.166	0.060	0.006	**Rural x Urbano**	-1.998	-1.801	-0.197	0.185	0.286
**R²**			0.999			**R²**			0.933			**R²**			0.966		
**ITEM 4**																	
**Rural x Urbano**	0.460	0.452	0.008	0.080	0.919	**Rural x Urbano**	0.461	0.432	0.028	0.081	0.724	**Rural x Urbano**	-1.538	-1.468	-0.070	0.122	0.568
**R²**			0.989			**R²**			0.996			**R²**			0.995		
**ITEM 5**																	
**Rural x Urbano**	2.406	2.427	-0.021	0.104	0.839	**Rural x Urbano**	2.401	2.548	-0.147	0.096	0.126	**Rural x Urbano**	0.934	0.887	0.047	0.102	0.648
**R²**			0.999			**R²**			0.961			**R²**			0.773		
**ITEM 6**																	
**Rural x Urbano**	1.588	1.613	-0.025	0.061	0.680	**Rural x Urbano**	1.711	1.708	0.003	0.080	0.972	**Rural x Urbano**	0.032	0.066	-0.034	0.058	0.551
**R²**			0.985			**R²**			0.998			**R²**			1.000		
**ITEM 7**																	
**Rural x Urbano**	2.972	2.959	0.014	0.180	0.940	**Rural x Urbano**	3.093	3.069	0.024	0.173	0.889	**Rural x Urbano**	1.404	1.268	0.135	0.102	0.184
**R²**			1.000			**R²**			1.000			**R²**			0.933		
**ITEM 8**																	
**Rural x Urbano**	2.781	2.691	0.090	0.125	0.470	**Rural x Urbano**	2.682	2.558	0.124	0.124	0.317	**Rural x Urbano**	1.393	1.256	0.137	0.115	0.231

**R^2**	0.999	**R^2**	0.999	**R^2**	0.966

Tabela S4 Cargas fatoriais não padronizadas do método de Alinhamento na *Escala Brasileira de Insegurança Alimentar* (versão reduzida, de 8 itens) de acordo com regiões brasileiras do domicílio, nas três partições. PNAD, 2013.

Partição 1						Partição 2						Partição 3					
**Grupos**	**Cargas fatoriais não padronizadas**		**Diferença**	**Erro-padrão da diferença**	**Valor de p**	**Grupos**	**Cargas fatoriais não padronizadas**		**Diferença**	**Erro-padrão da diferença**	**Valor de p**	**Grupos**	**Cargas fatoriais não padronizadas**		**Diferença**	**Erro-padrão da diferença**	**Valor de p**
**ITEM 1**																	
**NE x N**	3.045	2.699	0.346	0.194	0.075	**NE x N**	2.577	2.279	0.298	0.166	0.072	**NE x N**	***2.958***	***2.349***	***0.610***	***0.192***	***0.001***
**SE x N**	2.616	2.699	-0.083	0.188	0.657	**SE x N**	2.133	2.279	-0.146	0.170	0.391	**SE x N**	2.210	2.349	-0.139	0.169	0.412
**SE x NE**	2.616	3.045	-0.429	0.188	0.022	**SE x NE**	2.133	2.577	-0.445	0.169	0.008	**SE x NE**	***2.210***	***2.958***	***-0.748***	***0.186***	***0.000***
**S x N**	2.200	2.699	-0.499	0.215	0.020	**S x N**	1.982	2.279	-0.297	0.194	0.126	**S x N**	1.997	2.349	-0.352	0.191	0.065
**S x NE**	***2.200***	***3.045***	***-0.845***	***0.209***	***0.000***	**S x NE**	1.982	2.577	-0.595	0.191	0.002	**S x NE**	***1.997***	***2.958***	***-0.961***	***0.204***	***0.000***
**S x SE**	2.200	2.616	-0.416	0.208	0.045	**S x SE**	1.982	2.133	-0.151	0.192	0.431	**S x SE**	1.997	2.210	-0.213	0.187	0.256
**CO x N**	2.489	2.699	-0.210	0.246	0.392	**CO x N**	2.596	2.279	0.317	0.255	0.215	**CO x N**	2.346	2.349	-0.002	0.208	0.991
**CO x NE**	2.489	3.045	-0.556	0.244	0.022	**CO x NE**	2.596	2.577	0.018	0.238	0.939	**CO x NE**	2.346	2.958	-0.612	0.225	0.007
**CO x SE**	2.489	2.616	-0.127	0.231	0.582	**CO x SE**	2.596	2.133	0.463	0.257	0.071	**CO x SE**	2.346	2.210	0.136	0.211	0.518
**CO x S**	2.489	2.200	0.289	0.258	0.263	**CO x S**	2.596	1.982	0.614	0.273	0.025	**CO x S**	2.346	1.997	0.349	0.231	0.131
**R^2**			0.000			**R^2**			0.088			**R^2**			0.000		
**ITEM 2**																	
**NE x N**	3.543	3.504	0.040	0.202	0.845	**NE x N**	2.824	3.091	-0.267	0.193	0.165	**NE x N**	3.611	3.064	0.547	0.235	0.020
**SE x N**	2.882	3.504	-0.621	0.235	0.008	**SE x N**	2.686	3.091	-0.404	0.214	0.059	**SE x N**	2.852	3.064	-0.212	0.226	0.348
**SE x NE**	***2.882***	***3.543***	***-0.661***	***0.205***	***0.001***	**SE x NE**	2.686	2.824	-0.137	0.169	0.416	**SE x NE**	***2.852***	***3.611***	***-0.759***	***0.230***	***0.001***
**S x N**	***2.580***	***3.504***	***-0.923***	***0.256***	***0.000***	**S x N**	2.539	3.091	-0.551	0.252	0.029	**S x N**	2.920	3.064	-0.144	0.262	0.583
**S x NE**	***2.580***	***3.543***	***-0.963***	***0.230***	***0.000***	**S x NE**	2.539	2.824	-0.284	0.219	0.194	**S x NE**	2.920	3.611	-0.690	0.270	0.011
**S x SE**	2.580	2.882	-0.302	0.238	0.204	**S x SE**	2.539	2.686	-0.147	0.223	0.510	**S x SE**	2.920	2.852	0.069	0.257	0.789
**CO x N**	2.746	3.504	-0.757	0.267	0.005	**CO x N**	3.145	3.091	0.054	0.294	0.853	**CO x N**	3.275	3.064	0.211	0.339	0.534
**CO x NE**	***2.746***	***3.543***	***-0.797***	***0.244***	***0.001***	**CO x NE**	3.145	2.824	0.322	0.283	0.255	**CO x NE**	3.275	3.611	-0.336	0.336	0.318
**CO x SE**	2.746	2.882	-0.136	0.240	0.571	**CO x SE**	3.145	2.686	0.459	0.297	0.122	**CO x SE**	3.275	2.852	0.423	0.341	0.215
**CO x S**	2.746	2.580	0.166	0.265	0.531	**CO x S**	3.145	2.539	0.606	0.324	0.061	**CO x S**	3.275	2.920	0.354	0.370	0.338
**R^2**			0.000			**R^2**			0.434			**R^2**			0.000		
**ITEM 3**																	
**NE x N**	4.300	4.352	-0.053	0.321	0.870	**NE x N**	3.789	3.635	0.154	0.269	0.567	**NE x N**	3.886	3.804	0.083	0.282	0.770

**SE x N**	4.009	4.352	-0.344	0.383	0.369	**SE x N**	3.799	3.635	0.164	0.335	0.625	**SE x N**	3.947	3.804	0.143	0.361	0.692
**SE x NE**	4.009	4.300	-0.291	0.324	0.369	**SE x NE**	3.799	3.789	0.010	0.302	0.974	**SE x NE**	3.947	3.886	0.060	0.304	0.843
**S x N**	4.088	4.352	-0.264	0.510	0.605	**S x N**	3.826	3.635	0.191	0.411	0.643	**S x N**	3.879	3.804	0.075	0.362	0.836
**S x NE**	4.088	4.300	-0.211	0.471	0.654	**S x NE**	3.826	3.789	0.037	0.377	0.923	**S x NE**	3.879	3.886	-0.008	0.305	0.979
**S x SE**	4.088	4.009	0.080	0.496	0.872	**S x SE**	3.826	3.799	0.027	0.451	0.953	**S x SE**	3.879	3.947	-0.068	0.376	0.856
**CO x N**	4.474	4.352	0.121	0.451	0.788	**CO x N**	3.361	3.635	-0.274	0.396	0.489	**CO x N**	3.929	3.804	0.125	0.420	0.766
**CO x NE**	4.474	4.300	0.174	0.421	0.679	**CO x NE**	3.361	3.789	-0.428	0.373	0.251	**CO x NE**	3.929	3.886	0.042	0.369	0.908
**CO x SE**	4.474	4.009	0.465	0.474	0.326	**CO x SE**	3.361	3.799	-0.438	0.443	0.323	**CO x SE**	3.929	3.947	-0.018	0.427	0.966
**CO x S**	4.474	4.088	0.385	0.595	0.517	**CO x S**	3.361	3.826	-0.465	0.503	0.356	**CO x S**	3.929	3.879	0.050	0.428	0.906
**R^2**			0.750			**R^2**			0.833			**R^2**			0.985		
**ITEM 4**																	
**NE x N**	3.747	3.887	-0.140	0.255	0.585	**NE x N**	3.106	3.182	-0.076	0.217	0.728	**NE x N**	3.694	3.750	-0.055	0.237	0.815
**SE x N**	3.777	3.887	-0.110	0.289	0.703	**SE x N**	3.514	3.182	0.332	0.311	0.286	**SE x N**	3.711	3.750	-0.039	0.304	0.898
**SE x NE**	3.777	3.747	0.029	0.237	0.901	**SE x NE**	3.514	3.106	0.408	0.291	0.162	**SE x NE**	3.711	3.694	0.017	0.269	0.951
**S x N**	3.751	3.887	-0.136	0.394	0.731	**S x N**	3.449	3.182	0.267	0.388	0.492	**S x N**	3.766	3.750	0.017	0.325	0.959
**S x NE**	3.751	3.747	0.004	0.358	0.991	**S x NE**	3.449	3.106	0.342	0.375	0.361	**S x NE**	3.766	3.694	0.072	0.290	0.804
**S x SE**	3.751	3.777	-0.026	0.393	0.948	**S x SE**	3.449	3.514	-0.065	0.407	0.873	**S x SE**	3.766	3.711	0.055	0.349	0.874
**CO x N**	4.467	3.887	0.580	0.605	0.338	**CO x N**	3.547	3.182	0.366	0.333	0.272	**CO x N**	3.791	3.750	0.041	0.388	0.916
**CO x NE**	4.467	3.747	0.720	0.581	0.215	**CO x NE**	3.547	3.106	0.441	0.313	0.159	**CO x NE**	3.791	3.694	0.096	0.362	0.790
**CO x SE**	4.467	3.777	0.690	0.593	0.244	**CO x SE**	3.547	3.514	0.034	0.350	0.923	**CO x SE**	3.791	3.711	0.080	0.410	0.846
**CO x S**	4.467	3.751	0.716	0.665	0.282	**CO x S**	3.547	3.449	0.099	0.423	0.816	**CO x S**	3.791	3.766	0.024	0.417	0.954
**R^2**			0.670			**R^2**			0.902			**R^2**			0.992		
**ITEM 5**																	
**NE x N**	2.874	2.772	0.102	0.171	0.553	**NE x N**	2.888	2.801	0.087	0.165	0.599	**NE x N**	2.876	3.201	-0.325	0.209	0.119
**SE x N**	2.998	2.772	0.226	0.221	0.306	**SE x N**	3.030	2.801	0.229	0.230	0.321	**SE x N**	3.571	3.201	0.369	0.329	0.261
**SE x NE**	2.998	2.874	0.124	0.200	0.533	**SE x NE**	3.030	2.888	0.142	0.218	0.514	**SE x NE**	3.571	2.876	0.695	0.311	0.026
**S x N**	3.235	2.772	0.463	0.344	0.178	**S x N**	2.897	2.801	0.095	0.256	0.710	**S x N**	3.203	3.201	0.002	0.339	0.996
**S x NE**	3.235	2.874	0.361	0.333	0.278	**S x NE**	2.897	2.888	0.009	0.245	0.971	**S x NE**	3.203	2.876	0.327	0.336	0.331
**S x SE**	3.235	2.998	0.237	0.352	0.501	**S x SE**	2.897	3.030	-0.133	0.300	0.657	**S x SE**	3.203	3.571	-0.368	0.423	0.384
**CO x N**	3.241	2.772	0.469	0.394	0.235	**CO x N**	2.861	2.801	0.059	0.295	0.840	**CO x N**	2.802	3.201	-0.399	0.331	0.228

**CO x NE**	3.241	2.874	0.367	0.384	0.339	**CO x NE**	2.861	2.888	-0.027	0.289	0.925	**CO x NE**	2.802	2.876	-0.074	0.301	0.806
**CO x SE**	3.241	2.998	0.243	0.390	0.534	**CO x SE**	2.861	3.030	-0.169	0.335	0.614	**CO x SE**	2.802	3.571	-0.769	0.403	0.057
**CO x S**	3.241	3.235	0.006	0.429	0.989	**CO x S**	2.861	2.897	-0.036	0.353	0.919	**CO x S**	2.802	3.203	-0.401	0.426	0.346
**R^2**			0.803			**R^2**			0.957			**R^2**			0.677		
**ITEM 6**																	
**NE x N**	3.075	3.151	-0.076	0.149	0.610	**NE x N**	3.021	3.471	-0.450	0.210	0.032	**NE x N**	3.158	3.651	-0.494	0.237	0.037
**SE x N**	3.138	3.151	-0.013	0.167	0.939	**SE x N**	3.004	3.471	-0.467	0.251	0.064	**SE x N**	3.205	3.651	-0.446	0.274	0.104
**SE x NE**	3.138	3.075	0.063	0.142	0.656	**SE x NE**	3.004	3.021	-0.016	0.188	0.930	**SE x NE**	3.205	3.158	0.047	0.202	0.815
**S x N**	3.031	3.151	-0.120	0.222	0.587	**S x N**	3.294	3.471	-0.177	0.325	0.587	**S x N**	3.051	3.651	-0.601	0.317	0.058
**S x NE**	3.031	3.075	-0.044	0.201	0.826	**S x NE**	3.294	3.021	0.274	0.297	0.357	**S x NE**	3.051	3.158	-0.107	0.253	0.673
**S x SE**	3.031	3.138	-0.107	0.223	0.630	**S x SE**	3.294	3.004	0.290	0.325	0.372	**S x SE**	3.051	3.205	-0.154	0.289	0.593
**CO x N**	2.916	3.151	-0.235	0.251	0.349	**CO x N**	3.168	3.471	-0.303	0.344	0.379	**CO x N**	3.295	3.651	-0.356	0.357	0.319
**CO x NE**	2.916	3.075	-0.159	0.231	0.491	**CO x NE**	3.168	3.021	0.147	0.304	0.628	**CO x NE**	3.295	3.158	0.138	0.303	0.649
**CO x SE**	2.916	3.138	-0.222	0.242	0.359	**CO x SE**	3.168	3.004	0.164	0.329	0.619	**CO x SE**	3.295	3.205	0.090	0.330	0.784
**CO x S**	2.916	3.031	-0.115	0.276	0.678	**CO x S**	3.168	3.294	-0.126	0.392	0.747	**CO x S**	3.295	3.051	0.245	0.373	0.512
**R^2**			0.915			**R^2**			0.794			**R^2**			0.686		
**ITEM 7**																	
**NE x N**	3.386	3.552	-0.166	0.273	0.544	**NE x N**	3.536	3.537	-0.001	0.271	0.998	**NE x N**	4.004	3.882	0.121	0.312	0.698
**SE x N**	3.846	3.552	0.294	0.369	0.425	**SE x N**	3.192	3.537	-0.345	0.340	0.311	**SE x N**	4.013	3.882	0.130	0.394	0.741
**SE x NE**	3.846	3.386	0.460	0.344	0.181	**SE x NE**	3.192	3.536	-0.345	0.318	0.279	**SE x NE**	4.013	4.004	0.009	0.359	0.980
**S x N**	4.067	3.552	0.516	0.521	0.323	**S x N**	3.194	3.537	-0.343	0.384	0.372	**S x N**	4.285	3.882	0.402	0.551	0.465
**S x NE**	4.067	3.386	0.682	0.502	0.175	**S x NE**	3.194	3.536	-0.342	0.363	0.346	**S x NE**	4.285	4.004	0.281	0.521	0.589
**S x SE**	4.067	3.846	0.221	0.534	0.678	**S x SE**	3.194	3.192	0.003	0.382	0.995	**S x SE**	4.285	4.013	0.272	0.577	0.637
**CO x N**	3.339	3.552	-0.213	0.461	0.644	**CO x N**	3.133	3.537	-0.404	0.423	0.339	**CO x N**	3.753	3.882	-0.130	0.512	0.800
**CO x NE**	3.339	3.386	-0.047	0.429	0.913	**CO x NE**	3.133	3.536	-0.404	0.400	0.313	**CO x NE**	3.753	4.004	-0.251	0.495	0.612
**CO x SE**	3.339	3.846	-0.507	0.512	0.322	**CO x SE**	3.133	3.192	-0.059	0.412	0.886	**CO x SE**	3.753	4.013	-0.260	0.555	0.639
**CO x S**	3.339	4.067	-0.729	0.635	0.252	**CO x S**	3.133	3.194	-0.062	0.448	0.891	**CO x S**	3.753	4.285	-0.532	0.675	0.430
**R^2**			0.755			**R^2**			0.375			**R^2**			0.823		
**ITEM 8**																	
**NE x N**	2.569	2.440	0.129	0.177	0.465	**NE x N**	2.350	2.156	0.194	0.155	0.211	**NE x N**	2.472	2.777	-0.304	0.205	0.137
**SE x N**	3.175	2.440	0.735	0.273	0.007	**SE x N**	2.638	2.156	0.482	0.230	0.036	**SE x N**	2.987	2.777	0.210	0.263	0.424

**SE x NE**	3.175	2.569	0.606	0.264	0.022	**SE x NE**	2.638	2.350	0.288	0.218	0.186	**SE x NE**	2.987	2.472	0.514	0.245	0.036
**S x N**	3.601	2.440	1.161	0.473	0.014	**S x N**	2.945	2.156	0.789	0.322	0.014	**S x N**	3.837	2.777	1.060	0.478	0.027
**S x NE**	3.601	2.569	1.031	0.466	0.027	**S x NE**	2.945	2.350	0.595	0.313	0.057	**S x NE**	3.837	2.472	1.364	0.469	0.004
**S x SE**	3.601	3.175	0.426	0.500	0.394	**S x SE**	2.945	2.638	0.307	0.347	0.376	**S x SE**	3.837	2.987	0.850	0.498	0.088
**CO x N**	2.826	2.440	0.386	0.400	0.335	**CO x N**	2.323	2.156	0.167	0.259	0.518	**CO x N**	3.276	2.777	0.499	0.444	0.261
**CO x NE**	2.826	2.569	0.257	0.393	0.513	**CO x NE**	2.323	2.350	-0.026	0.246	0.914	**CO x NE**	3.276	2.472	0.804	0.427	0.060
**CO x SE**	2.826	3.175	-0.349	0.433	0.420	**CO x SE**	2.323	2.638	-0.315	0.305	0.303	**CO x SE**	3.276	2.987	0.289	0.457	0.527
**CO x S**	2.826	3.601	-0.775	0.568	0.173	**CO x S**	2.323	2.945	-0.622	0.379	0.101	**CO x S**	3.276	3.837	-0.561	0.612	0.359
**R^2**			0.573			**R^2**			0.694			**R^2**			0.568		

Legenda: N = Norte; NE = Nordeste; SE = Sudeste; S = Sul; CO = Centro-Oeste.

$p \leq 0,001$

Tabela S5 Limiares do método de Alinhamento na *Escala Brasileira de Insegurança Alimentar* (versão reduzida, de 8 itens) de acordo com regiões brasileiras do domicílio, nas três partições. PNAD, 2013.

Partição 1						Partição 2						Partição 3					
**Grupos**	**Limiares**		**Diferença**	**Erro-padrão da diferença**	**Valor de p**	**Grupos**	**Limiares**		**Diferença**	**Erro-padrão da diferença**	**Valor de p**	**Grupos**	**Limiares**		**Diferença**	**Erro-padrão da diferença**	**Valor de p**
									**ITEM 1**								
**NE x N**	-0.786	-0.739	-0.047	0.074	0.523	**NE x N**	-0.496	-0.366	-0.130	0.086	0.131	**NE x N**	-0.702	-0.643	-0.059	0.077	0.449
**SE x N**	-0.863	-0.739	-0.124	0.086	0.151	**SE x N**	-0.556	-0.366	-0.190	0.072	0.008	**SE x N**	-0.638	-0.643	0.005	0.072	0.941
**SE x NE**	-0.863	-0.786	-0.077	0.082	0.348	**SE x NE**	-0.556	-0.496	-0.060	0.091	0.508	**SE x NE**	-0.638	-0.702	0.064	0.077	0.406
**S x N**	-0.790	-0.739	-0.052	0.087	0.554	**S x N**	-0.433	-0.366	-0.067	0.081	0.411	**S x N**	-0.520	-0.643	0.123	0.090	0.171
**S x NE**	-0.790	-0.786	-0.004	0.084	0.961	**S x NE**	-0.433	-0.496	0.063	0.103	0.538	**S x NE**	-0.520	-0.702	0.182	0.099	0.066
**S x SE**	-0.790	-0.863	0.073	0.098	0.458	**S x SE**	-0.433	-0.556	0.124	0.089	0.166	**S x SE**	-0.520	-0.638	0.118	0.093	0.205
**CO x N**	-0.827	-0.739	-0.088	0.101	0.381	**CO x N**	-0.689	-0.366	-0.323	0.102	0.002	**CO x N**	-0.669	-0.643	-0.026	0.094	0.781
**CO x NE**	-0.827	-0.786	-0.041	0.098	0.674	**CO x NE**	-0.689	-0.496	-0.193	0.090	0.032	**CO x NE**	-0.669	-0.702	0.032	0.097	0.738
**CO x SE**	-0.827	-0.863	0.036	0.109	0.742	**CO x SE**	-0.689	-0.556	-0.132	0.103	0.197	**CO x SE**	-0.669	-0.638	-0.031	0.095	0.741
**CO x S**	-0.827	-0.790	-0.037	0.112	0.741	**CO x S**	-0.689	-0.433	-0.256	0.112	0.022	**CO x S**	-0.669	-0.520	-0.149	0.110	0.175
**R²**			0.860			**R²**			0.901			**R²**			0.902		
**ITEM 2**																	
**NE x N**	-0.124	-0.131	0.006	0.061	0.917	**NE x N**	0.148	0.191	-0.043	0.061	0.483	**NE x N**	-0.035	0.029	-0.064	0.073	0.382
**SE x N**	0.058	-0.131	0.189	0.091	0.038	**SE x N**	0.345	0.191	0.154	0.077	0.045	**SE x N**	0.121	0.029	0.092	0.068	0.173
**SE x NE**	0.058	-0.124	0.182	0.085	0.031	**SE x NE**	0.345	0.148	0.197	0.067	0.003	**SE x NE**	0.121	-0.035	0.156	0.081	0.053
**S x N**	0.071	-0.131	0.202	0.100	0.043	**S x N**	0.380	0.191	0.189	0.099	0.057	**S x N**	0.182	0.029	0.153	0.080	0.055
**S x NE**	0.071	-0.124	0.196	0.094	0.037	**S x NE**	0.380	0.148	0.232	0.083	0.005	**S x NE**	0.182	-0.035	0.217	0.085	0.011
**S x SE**	0.071	0.058	0.013	0.088	0.880	**S x SE**	0.380	0.345	0.035	0.089	0.691	**S x SE**	0.182	0.121	0.060	0.082	0.461
**CO x N**	-0.038	-0.131	0.093	0.106	0.383	**CO x N**	0.250	0.191	0.059	0.086	0.490	**CO x N**	-0.055	0.029	-0.084	0.098	0.392
**CO x NE**	-0.038	-0.124	0.086	0.102	0.397	**CO x NE**	0.250	0.148	0.102	0.089	0.255	**CO x NE**	-0.055	-0.035	-0.020	0.087	0.821
**CO x SE**	-0.038	0.058	-0.096	0.101	0.340	**CO x SE**	0.250	0.345	-0.095	0.100	0.343	**CO x SE**	-0.055	0.121	-0.176	0.103	0.087
**CO x S**	-0.038	0.071	-0.109	0.107	0.309	**CO x S**	0.250	0.380	-0.130	0.121	0.282	**CO x S**	-0.055	0.182	-0.236	0.108	0.029
**R²**			0.869			**R²**			0.956			**R²**			0.967		
**ITEM 3**																	
**NE x N**	-0.487	-0.385	-0.102	0.087	0.240	**NE x N**	***-0.184***	***0.145***	***-0.329***	***0.087***	***0.000***	**NE x N**	-0.301	-0.226	-0.074	0.072	0.300

**SE x N**	-0.570	-0.385	-0.184	0.114	0.107	**SE x N**	***-0.263***	***0.145***	***-0.408***	***0.116***	***0.000***	**SE x N**	-0.534	-0.226	-0.308	0.104	0.003
**SE x NE**	-0.570	-0.487	-0.082	0.100	0.411	**SE x NE**	-0.263	-0.184	-0.078	0.097	0.419	**SE x NE**	-0.534	-0.301	-0.234	0.095	0.014
**S x N**	-0.661	-0.385	-0.275	0.150	0.067	**S x N**	-0.251	0.145	-0.396	0.139	0.004	**S x N**	-0.392	-0.226	-0.166	0.118	0.161
**S x NE**	-0.661	-0.487	-0.174	0.141	0.220	**S x NE**	-0.251	-0.184	-0.066	0.114	0.560	**S x NE**	-0.392	-0.301	-0.091	0.108	0.399
**S x SE**	-0.661	-0.570	-0.091	0.165	0.581	**S x SE**	-0.251	-0.263	0.012	0.132	0.928	**S x SE**	-0.392	-0.534	0.142	0.123	0.248
**CO x N**	-0.925	-0.385	-0.540	0.190	0.005	**CO x N**	-0.108	0.145	-0.253	0.114	0.026	**CO x N**	-0.493	-0.226	-0.266	0.124	0.032
**CO x NE**	-0.925	-0.487	-0.438	0.186	0.019	**CO x NE**	-0.108	-0.184	0.076	0.129	0.555	**CO x NE**	-0.493	-0.301	-0.192	0.117	0.100
**CO x SE**	-0.925	-0.570	-0.355	0.203	0.080	**CO x SE**	-0.108	-0.263	0.154	0.152	0.310	**CO x SE**	-0.493	-0.534	0.042	0.131	0.751
**CO x S**	-0.925	-0.661	-0.264	0.216	0.221	**CO x S**	-0.108	-0.251	0.142	0.170	0.401	**CO x S**	-0.493	-0.392	-0.101	0.142	0.478
**R^2**			0.975			**R^2**				0.982		**R^2**			0.999		
**ITEM 4**																	
**NE x N**	-0.139	0.090	-0.229	0.072	0.002	**NE x N**	***0.169***	***0.401***	***-0.232***	***0.061***	***0.000***	**NE x N**	-0.027	0.129	-0.156	0.063	0.013
**SE x N**	***-0.232***	***0.090***	***-0.322***	***0.089***	***0.000***	**SE x N**	0.124	0.401	-0.277	0.120	0.021	**SE x N**	***-0.141***	***0.129***	***-0.270***	***0.084***	***0.001***
**SE x NE**	-0.232	-0.139	-0.093	0.080	0.244	**SE x NE**	0.124	0.169	-0.046	0.109	0.676	**SE x NE**	-0.141	-0.027	-0.114	0.080	0.157
**S x N**	***-0.356***	***0.090***	***-0.446***	***0.126***	***0.000***	**S x N**	0.043	0.401	-0.358	0.125	0.004	**S x N**	***-0.218***	***0.129***	***-0.347***	***0.094***	***0.000***
**S x NE**	-0.356	-0.139	-0.217	0.121	0.073	**S x NE**	0.043	0.169	-0.126	0.113	0.267	**S x NE**	-0.218	-0.027	-0.191	0.088	0.030
**S x SE**	-0.356	-0.232	-0.124	0.130	0.341	**S x SE**	0.043	0.124	-0.080	0.106	0.451	**S x SE**	-0.218	-0.141	-0.077	0.099	0.437
**CO x N**	***-0.621***	***0.090***	***-0.710***	***0.182***	***0.000***	**CO x N**	0.076	0.401	-0.325	0.117	0.006	**CO x N**	***-0.294***	***0.129***	***-0.423***	***0.112***	***0.000***
**CO x NE**	-0.621	-0.139	-0.482	0.189	0.011	**CO x NE**	0.076	0.169	-0.093	0.106	0.378	**CO x NE**	-0.294	-0.027	-0.267	0.109	0.014
**CO x SE**	-0.621	-0.232	-0.388	0.187	0.037	**CO x SE**	0.076	0.124	-0.048	0.103	0.642	**CO x SE**	-0.294	-0.141	-0.154	0.117	0.190
**CO x S**	-0.621	-0.356	-0.264	0.197	0.180	**CO x S**	0.076	0.043	0.033	0.115	0.777	**CO x S**	-0.294	-0.218	-0.076	0.124	0.537
**R^2**			0.963			**R^2**				0.994		**R^2**			0.993		
**ITEM 5**																	
**NE x N**	1.960	1.754	0.207	0.093	0.026	**NE x N**	***2.410***	***1.983***	***0.427***	***0.123***	***0.001***	**NE x N**	2.040	1.967	0.072	0.098	0.462
**SE x N**	1.928	1.754	0.174	0.111	0.117	**SE x N**	2.371	1.983	0.388	0.148	0.009	**SE x N**	2.209	1.967	0.242	0.132	0.067
**SE x NE**	1.928	1.960	-0.032	0.098	0.743	**SE x NE**	2.371	2.410	-0.039	0.135	0.773	**SE x NE**	2.209	2.040	0.169	0.125	0.175
**S x N**	2.037	1.754	0.283	0.156	0.070	**S x N**	2.250	1.983	0.267	0.195	0.169	**S x N**	1.968	1.967	0.001	0.147	0.995
**S x NE**	2.037	1.960	0.077	0.145	0.597	**S x NE**	2.250	2.410	-0.160	0.181	0.377	**S x NE**	1.968	2.040	-0.071	0.138	0.603
**S x SE**	2.037	1.928	0.109	0.157	0.487	**S x SE**	2.250	2.371	-0.121	0.195	0.537	**S x SE**	1.968	2.209	-0.241	0.176	0.171
**CO x N**	2.232	1.754	0.478	0.210	0.023	**CO x N**	2.688	1.983	0.706	0.260	0.007	**CO x N**	1.997	1.967	0.030	0.152	0.843

**CO x NE**	2.232	1.960	0.272	0.203	0.182	**CO x NE**	2.688	2.410	0.279	0.260	0.283	**CO x NE**	1.997	2.040	-0.042	0.148	0.775
**CO x SE**	2.232	1.928	0.304	0.213	0.154	**CO x SE**	2.688	2.371	0.318	0.280	0.257	**CO x SE**	1.997	2.209	-0.212	0.170	0.213
**CO x S**	2.232	2.037	0.195	0.242	0.421	**CO x S**	2.688	2.250	0.438	0.302	0.146	**CO x S**	1.997	1.968	0.029	0.180	0.871
**R^2**			0.948			**R^2**			0.975			**R^2**			0.914		
**ITEM 6**																	
**NE x N**	1.111	1.191	-0.080	0.063	0.209	**NE x N**	1.397	1.611	-0.214	0.094	0.023	**NE x N**	1.254	1.329	-0.075	0.089	0.399
**SE x N**	1.072	1.191	-0.119	0.074	0.107	**SE x N**	1.453	1.611	-0.158	0.105	0.133	**SE x N**	1.259	1.329	-0.070	0.092	0.448
**SE x NE**	1.072	1.111	-0.040	0.062	0.525	**SE x NE**	1.453	1.397	0.055	0.087	0.523	**SE x NE**	1.259	1.254	0.005	0.077	0.947
**S x N**	0.982	1.191	-0.209	0.095	0.028	**S x N**	1.562	1.611	-0.049	0.121	0.686	**S x N**	1.159	1.329	-0.170	0.109	0.119
**S x NE**	0.982	1.111	-0.129	0.086	0.134	**S x NE**	1.562	1.397	0.165	0.123	0.181	**S x NE**	1.159	1.254	-0.095	0.088	0.277
**S x SE**	0.982	1.072	-0.090	0.093	0.336	**S x SE**	1.562	1.453	0.109	0.134	0.413	**S x SE**	1.159	1.259	-0.100	0.102	0.327
**CO x N**	1.285	1.191	0.094	0.111	0.399	**CO x N**	1.856	1.611	0.245	0.164	0.134	**CO x N**	1.502	1.329	0.173	0.131	0.186
**CO x NE**	1.285	1.111	0.173	0.105	0.100	**CO x NE**	1.856	1.397	0.459	0.147	0.002	**CO x NE**	1.502	1.254	0.248	0.119	0.038
**CO x SE**	1.285	1.072	0.213	0.114	0.061	**CO x SE**	1.856	1.453	0.403	0.159	0.011	**CO x SE**	1.502	1.259	0.243	0.128	0.058
**CO x S**	1.285	0.982	0.303	0.129	0.019	**CO x S**	1.856	1.562	0.294	0.183	0.109	**CO x S**	1.502	1.159	0.344	0.137	0.012
**R^2**			0.992			**R^2**			0.969			**R^2**			0.954		
**ITEM 7**																	
**NE x N**	2.334	2.358	-0.023	0.144	0.872	**NE x N**	2.964	2.677	0.287	0.173	0.098	**NE x N**	2.928	2.547	0.381	0.198	0.054
**SE x N**	2.476	2.358	0.119	0.171	0.488	**SE x N**	2.919	2.677	0.242	0.220	0.272	**SE x N**	2.812	2.547	0.265	0.227	0.244
**SE x NE**	2.476	2.334	0.142	0.160	0.374	**SE x NE**	2.919	2.964	-0.045	0.213	0.833	**SE x NE**	2.812	2.928	-0.116	0.219	0.595
**S x N**	2.382	2.358	0.024	0.232	0.917	**S x N**	2.713	2.677	0.036	0.228	0.875	**S x N**	2.606	2.547	0.059	0.278	0.832
**S x NE**	2.382	2.334	0.048	0.231	0.837	**S x NE**	2.713	2.964	-0.251	0.225	0.265	**S x NE**	2.606	2.928	-0.322	0.282	0.254
**S x SE**	2.382	2.476	-0.094	0.251	0.707	**S x SE**	2.713	2.919	-0.206	0.269	0.444	**S x SE**	2.606	2.812	-0.206	0.299	0.491
**CO x N**	2.458	2.358	0.101	0.259	0.698	**CO x N**	2.680	2.677	0.003	0.263	0.992	**CO x N**	2.804	2.547	0.257	0.278	0.356
**CO x NE**	2.458	2.334	0.124	0.255	0.627	**CO x NE**	2.680	2.964	-0.284	0.261	0.276	**CO x NE**	2.804	2.928	-0.125	0.270	0.644
**CO x SE**	2.458	2.476	-0.018	0.267	0.946	**CO x SE**	2.680	2.919	-0.240	0.302	0.427	**CO x SE**	2.804	2.812	-0.008	0.291	0.978
**CO x S**	2.458	2.382	0.076	0.314	0.808	**CO x S**	2.680	2.713	-0.033	0.312	0.915	**CO x S**	2.804	2.606	0.198	0.341	0.561
**R^2**			0.953			**R^2**			0.972			**R^2**			0.981		
**ITEM 8**																	
**NE x N**	***2.362***	***1.996***	***0.366***	***0.109***	***0.001***	**NE x N**	***2.518***	***2.093***	***0.424***	***0.126***	***0.001***	**NE x N**	2.356	2.256	0.100	0.119	0.400
**SE x N**	2.325	1.996	0.329	0.151	0.029	**SE x N**	2.524	2.093	0.430	0.160	0.007	**SE x N**	2.236	2.256	-0.020	0.138	0.883

**SE x NE**	2.325	2.362	-0.037	0.135	0.786	**SE x NE**	2.524	2.518	0.006	0.135	0.964	**SE x NE**	2.236	2.356	-0.121	0.134	0.369
**S x N**	2.414	1.996	0.418	0.176	0.018	**S x N**	2.578	2.093	0.485	0.170	0.004	**S x N**	2.574	2.256	0.318	0.199	0.110
**S x NE**	2.414	2.362	0.052	0.162	0.746	**S x NE**	2.578	2.518	0.061	0.150	0.687	**S x NE**	2.574	2.356	0.218	0.182	0.231
**S x SE**	2.414	2.325	0.089	0.201	0.657	**S x SE**	2.578	2.524	0.054	0.190	0.775	**S x SE**	2.574	2.236	0.338	0.208	0.104
**CO x N**	2.383	1.996	0.386	0.230	0.092	**CO x N**	2.441	2.093	0.347	0.205	0.091	**CO x N**	2.356	2.256	0.528	0.280	0.060
**CO x NE**	2.383	2.362	0.021	0.218	0.925	**CO x NE**	2.441	2.518	-0.077	0.187	0.680	**CO x NE**	2.356	2.356	0.428	0.275	0.120
**CO x SE**	2.383	2.325	0.057	0.232	0.805	**CO x SE**	2.441	2.524	-0.083	0.202	0.681	**CO x SE**	2.356	2.236	0.549	0.285	0.055
**CO x S**	2.383	2.414	-0.032	0.260	0.902	**CO x S**	2.441	2.578	0.137	0.211	0.515	**CO x S**	2.356	2.574	0.210	0.326	0.519
**R^2**			0.875			**R^2**			0.902			**R^2**			0.853		

Legenda: N = Norte; NE = Nordeste; SE = Sudeste; S = Sul; CO = Centro-Oeste.

$p \leq 0{,}001$

Tabela S6 Cargas fatoriais não padronizadas do método de Alinhamento na *Escala Brasileira de Insegurança Alimentar* (versão reduzida, de 8 itens) de acordo com as Unidades da Federação do domicílio, por partição. PNAD, 2013

**Partição 1**					**Partição 2**					**Partição 3**				
**Grupos**	**Cargas não padronizadas**	**Diferença**	**Erro-padrão da diferença**	**Valor de p**	**Grupos**	**Cargas não padronizadas**	**Diferença**	**Erro-padrão da diferença**	**Valor de p**	**Grupos**	**Cargas não padronizadas**	**Diferença**	**Erro-padrão da diferença**	**Valor de p**
**ITEM 1**														
**R^2**		0.331			**R^2**		0.379			**R^2**		0.162		
**ITEM 2**														
**R^2**		0.243			**R^2**		0.525			**R^2**		0.603		
**ITEM 3**														
**R^2**		0.268			**R^2**		0.280			**R^2**		0.752		
**ITEM 4**														
**R^2**		0.251			**R^2**		0.648			**R^2**		0.770		
**ITEM 5**														
**R^2**		0.838			**R^2**		0.851			**R^2**		0.658		
**ITEM 6**														
										**PB x AM**	***2.198 4.274***	***-2.076***	***0.557***	***0.000***
										**PB x PA**	***2.198 3.979***	***-1.781***	***0.470***	***0.000***
										**PB x MA**	***2.198 4.450***	***-2.252***	***0.637***	***0.000***
										**PB x CE**	***2.198 3.806***	***-1.608***	***0.478***	***0.001***
										**PE x PB**	***3.911 2.198***	***1.713***	***0.499***	***0.001***
										**BA x PB**	***3.812 2.198***	***1.614***	***0.464***	***0.001***
										**MG x PB**	***3.901 2.198***	***1.703***	***0.479***	***0.000***
										**RJ x PB**	***4.043 2.198***	***1.845***	***0.578***	***0.001***
										**SP x PB**	***3.963 2.198***	***1.765***	***0.553***	***0.001***
**R^2**		0.606			**R^2**		0.647			**R^2**		0.583		
**ITEM 7**														
**R^2**		0.744			**R^2**		0.434			**R^2**		0.261		
**ITEM 8**														
**R^2**		0.744			**R^2**		0.474			**R^2**		0.589		

Legenda: AM = Amazonas; RR = Roraima; PA = Pará; AP = Amapá; TO = Tocantins; MA = Maranhão; PI = Piauí; CE = Ceará; RN = Rio Grande do Norte; PB = Paraíba; PE = Pernambuco; SE = Sergipe; BA = Bahia; MG = Minas Gerais; ES = Espírito Santo; RJ = Rio de Janeiro; SP = São Paulo; PR = Paraná; SC = Santa Catarina; RS = Rio Grande do Sul; MS = Mato Grosso do Sul; MT = Mato Grosso; GO = Goiás; DF = Distrito Federal.

$p \leq 0,001$

Tabela S7 Limiares do método de Alinhamento na *Escala Brasileira de Insegurança Alimentar* (versão reduzida, de 8 itens) de acordo com as Unidades da Federação do domicílio, nas três partições. PNAD, 2013

**Partição 1**						**Partição 2**						**Partição 3**					
**Grupos**	**Limiares**		**Diferença**	**Erro-padrão da diferença**	**Valor de p**	**Grupos**	**Limiares**		**Diferença**	**Erro-padrão da diferença**	**Valor de p**	**Grupos**	**Limiares**		**Diferença**	**Erro-padrão da diferença**	**Valor de p**
**ITEM 1**																	
**R^2**			0.697			**R^2**			0.722			**R^2**			0.584		
**ITEM 2**																	
						**SP x MA**	***0.122***	***-0.327***	***0.449***	***0.138***	***0.001***						
**SC x TO**	***0.284***	***-0.857***	***1.141***	***0.316***	***0.000***												
**SC x MA**	***0.284***	***-0.531***	***0.815***	***0.235***	***0.001***												
**SC x PI**	***0.284***	***-0.534***	***0.818***	***0.241***	***0.001***												
**SC x CE**	***0.284***	***-0.470***	***0.754***	***0.220***	***0.001***												
**SC x PB**	***0.284***	***-0.967***	***1.250***	***0.369***	***0.001***												
**SC x BA**	***0.284***	***-0.365***	***0.649***	***0.202***	***0.001***												
						**RS x MA**	***0.170***	***-0.327***	***0.497***	***0.142***	***0.000***						
**R^2**			0.636			**R^2**			0.855			**R^2**			0.836		
**ITEM 3**																	
**TO x AM**	***-1.507***	***-0.202***	***-1.305***	***0.366***	***0.000***							TO x AM	***-1.017***	***-0.082***	***-0.935***	***0.271***	***0.001***
						**MA x AM**	***-0.886***	***-0.042***	***-0.845***	***0.209***	***0.000***						
**CE x AM**	***-1.009***	***-0.202***	***-0.807***	***0.227***	***0.000***	**CE x AM**	***-0.631***	***-0.042***	***-0.590***	***0.181***	***0.001***						
**PE x AM**	***-1.290***	***-0.202***	***-1.089***	***0.271***	***0.000***												
						**BA x AM**	***-0.630***	***-0.042***	***-0.588***	***0.159***	***0.000***						
						**MG x AM**	***-1.027***	***-0.042***	***-0.985***	***0.240***	***0.000***	**MG x AM**	***-0.715***	***-0.082***	***-0.634***	***0.198***	***0.001***
						**MG x PA**	***-1.027***	***-0.339***	***-0.688***	***0.206***	***0.001***						
						**RJ x AM**	***-0.906***	***-0.042***	***-0.864***	***0.265***	***0.001***						
**PR x AM**	***-1.300***	***-0.202***	***-1.098***	***0.301***	***0.000***	**PR x AM**	***-1.030***	***-0.042***	***-0.988***	***0.303***	***0.001***						
**MS x AM**	***-1.261***	***-0.202***	***-1.060***	***0.294***	***0.000***												
**GO x AM**	***-1.300***	***-0.202***	***-1.099***	***0.332***	***0.001***												
**DF x AM**	***-1.109***	***-0.202***	***-0.907***	***0.276***	***0.001***												
**R^2**			0.800			**R^2**			0.775			**R^2**			0.951		

**ITEM 4**																	
**RR x AM**	-0.744	0.199	-0.943	0.289	0.001												
												**TO x AM**	-0.678	0.228	-0.905	0.199	0.000
**MA x AM**	-0.382	0.199	-0.581	0.174	0.001												
												**MA x TO**	-0.024	-0.678	0.653	0.193	0.001
**PI x AM**	-0.679	0.199	-0.879	0.192	0.000												
												**CE x TO**	-0.027	-0.678	0.651	0.189	0.001
**PB x AM**	-0.860	0.199	-1.059	0.278	0.000												
**PE x AM**	-0.523	0.199	-0.723	0.198	0.000												
												**PE x TO**	0.041	-0.678	0.718	0.209	0.001
**BA x AM**	-0.579	0.199	-0.779	0.154	0.000	**BA x AM**	-0.314	0.192	-0.505	0.139	0.000	**BA x AM**	-0.342	0.228	-0.569	0.156	0.000
**MG x AM**	-0.546	0.199	-0.745	0.177	0.000												
												**ES x TO**	0.290	-0.678	0.967	0.259	0.000
**RJ x AM**	-0.818	0.199	-1.017	0.213	0.000	**RJ x AM**	-0.572	0.192	-0.764	0.237	0.001						
**SP x AM**	-0.496	0.199	-0.695	0.183	0.000												
**PR x AM**	-0.800	0.199	-0.999	0.232	0.000	**PR x AM**	-0.667	0.192	-0.859	0.235	0.000	**PR x AM**	-0.438	0.228	-0.665	0.195	0.001
												**SC x AM**	-0.561	0.228	-0.789	0.240	0.001
**RS x AM**	-0.652	0.199	-0.851	0.223	0.000												
**MS x AM**	-0.880	0.199	-1.079	0.290	0.000												
						**MT x AM**	-0.599	0.192	-0.791	0.238	0.001						
**GO x AM**	-1.031	0.199	-1.230	0.305	0.000	**GO x AM**	-0.426	0.192	-0.618	0.192	0.001	**GO x AM**	-0.925	0.228	-1.153	0.318	0.000
												**GO x RR**	-0.925	0.298	-1.223	0.373	0.001
												**GO x PE**	-0.925	0.041	-0.966	0.299	0.001
												**GO x ES**	-0.925	0.290	-1.215	0.337	0.000
**R^2**			0.839			**R^2**				0.865		**R^2**				0.934	
**ITEM 5**																	
						**SE x AM**	2.894	1.553	1.341	0.389	0.001						
						**SE x PA**	2.894	1.579	1.315	0.369	0.000						
**R^2**			0.921			**R^2**				0.912		**R^2**				0.769	
**ITEM 6**																	

**PA x AM**	0.815	1.328	-0.512	0.147	0.000												
						**PI x AP**	0.655	1.660	-1.005	0.288	0.000						
						**PI x MA**	0.655	1.350	-0.695	0.198	0.000						
**CE x AM**	0.844	1.328	-0.484	0.149	0.001												
**RN x AM**	0.654	1.328	-0.674	0.185	0.000												
**SE x AM**	0.465	1.328	-0.863	0.192	0.000												
**BA x AM**	0.688	1.328	-0.640	0.144	0.000												
												**BA x MA**	0.902	1.391	-0.489	0.151	0.001
												**MG x BA**	1.331	0.902	0.430	0.125	0.001
**ES x SE**	1.328	0.465	0.862	0.249	0.001												
**RJ x AM**	0.763	1.328	-0.565	0.167	0.001												
**SP x AM**	0.804	1.328	-0.524	0.164	0.001												
**SC x AM**	0.729	1.328	-0.598	0.188	0.001												
**RS x AM**	0.685	1.328	-0.643	0.172	0.000												
						**MT x PI**	1.712	0.655	1.057	0.313	0.001						
						**GO x PI**	1.335	0.655	0.680	0.205	0.001						
**GO x SE**	1.170	0.465	0.705	0.216	0.001												
**R^2**			0.818			**R^2**				0.868		**R^2**			0.873		
**ITEM 7**																	
**R^2**			0.898			**R^2**				0.822		**R^2**			0.763		
**ITEM 8**																	
						**BA x AM**	2.403	1.575	0.828	0.237	0.000						
						**BA x RR**	2.403	1.470	0.932	0.287	0.001						
**R^2**			0.864			**R^2**				0.694		**R^2**			0.859		

Legenda: AM = Amazonas; RR = Roraima; PA = Pará; AP = Amapá; TO = Tocantins; MA = Maranhão; PI = Piauí; CE = Ceará; RN = Rio Grande do Norte; PB = Paraíba; PE = Pernambuco; SE = Sergipe; BA = Bahia; MG = Minas Gerais; ES = Espírito Santo; RJ = Rio de Janeiro; SP = São Paulo; PR = Paraná; SC = Santa Catarina; RS = Rio Grande do Sul; MS = Mato Grosso do Sul; MT = Mato Grosso; GO = Goiás; DF = Distrito Federal.

$p \leq 0,001$

Figura S1 Síntese da análise de invariância da *Escala Brasileira de Insegurança Alimentar* (versão reduzida, de 8 itens) pelo método de Alinhamento, segundo perfil específico do Estado do Amazonas, por partição. PNAD, 2013

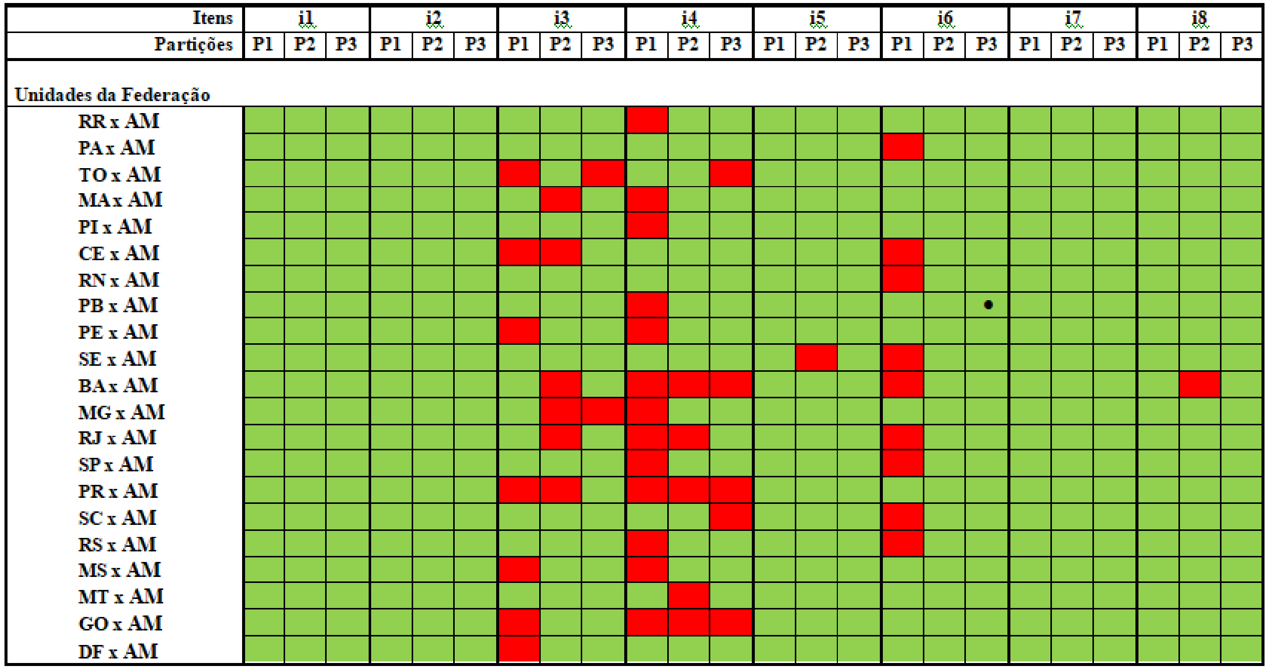

AM = Amazonas; RR = Roraima; PA = Pará; AP = Amapá; TO = Tocantins; MA = Maranhão; PI = Piauí; CE = Ceará; RN = Rio Grande do Norte; PB = Paraíba; PE = Pernambuco; SE = Sergipe; BA = Bahia; MG = Minas Gerais; RJ = Rio de Janeiro; SP = São Paulo; PR = Paraná; SC = Santa Catarina; RS = Rio Grande do Sul; MS = Mato Grosso do Sul; MT = Mato Grosso; GO = Goiás; DF = Distrito Federal.

Legenda: (verde) = invariância (carga e limiar);
(verde com ponto) = violação de invariância de carga;
(vermelho) = violação de invariância de limiar;
(vermelho com ponto) = violação de invariância de carga e limiar.
